# Development of the Emoji Faces Pain Scale and Its Validation on Mobile Devices in Adult Surgery Patients: Longitudinal Observational Study

**DOI:** 10.2196/41189

**Published:** 2023-04-17

**Authors:** Lili Li, Sicheng Wu, Jian Wang, Chunchun Wang, Weixin Zuo, Liping Yu, Jiangang Song

**Affiliations:** 1 Anesthesiology Department Shuguang Hospital Shanghai University of Traditional Chinese Medicine Shanghai China; 2 Faculty of Dentistry The University of Hong Kong Hong Kong SAR China (Hong Kong); 3 Acupuncture and Anesthesia Research Institute Shuguang Hospital Shanghai University of Traditional Chinese Medicine Shanghai China

**Keywords:** pain, mHealth, scale development, emoji, surgery

## Abstract

**Background:**

Measuring pain on digital devices using classic unidimensional pain scales such as the visual analog scale (VAS), numerical rating scale (NRS), and faces pain scale (FPS) has been proven to be reliable and valid. Emoji are pictographs designed in colorful form following the Unicode standard. It could be more beneficial to use emoji as faces of FPS on digital devices because emoji can easily fit on most devices and emoji are open-source so no approval would be needed before use. With a concise and user-friendly design, the emoji faces pain scale (Emoji-FPS) might be more generalizable to a wider population and more preferred by digital device users.

**Objective:**

This study was designed to develop an Emoji-FPS as well as to evaluate its reliability, validity, and preference on mobile devices in adult patients who underwent surgery.

**Methods:**

A modified Delphi technique with 2 rounds of web-based surveys was applied to obtain panelists’ consensus on the sequence of emoji that can best represent 6 levels of pain. The initial candidate sequences of emoji for the Delphi process were constructed referring to 2 well-validated FPSs (Wong-Baker FACES pain rating scale [Wong-Baker FACES] and faces pain scale-revised [FPS-R]). Then, a prospective cohort of patients scheduled to receive perianal surgery was recruited and asked to complete a web-based questionnaire on a mobile device at 5 time points (before surgery [T1], wake up after surgery [T2], 4 hours after surgery [T3], the second day after surgery [T4], and 15 minutes after T4 [T5]). The 4 well-validated pain scales (NRS, VAS, Wong-Baker FACES, and FPS-R) were used as reference scales.

**Results:**

After 2 rounds of surveys on 40 Delphi panelists, an Emoji-FPS was finally determined to represent 6 pain levels (0, 2, 4, 6, 8, and 10) from “no hurt” to “hurts worst.” For validation, 300 patients were recruited and 299 were analyzed, the mean age of whom was 38.5 (SD 10.5) years, and 106 (35.5%) were women. For concurrent validity, the Emoji-FPS was highly correlated with 4 reference scales with Spearman correlation coefficient ρ ranging from 0.91 to 0.95. Excellent agreements were observed between 4 versions of Emoji-FPS (iOS, Android, Microsoft, and OpenMoji), with weighted κ coefficients ranging from 0.96 to 0.97. For discriminant validity, patients’ mean preoperative Emoji-FPS score (T1) was significantly higher than their postoperative Emoji-FPS score (T4) with a difference of 1.4 (95% CI 1.3-1.6; *P*<.001). For test-retest reliability, Emoji-FPS scores measured at T4 and T5 were highly correlated with a ρ of 0.91. The Emoji-FPS was mostly preferred, followed by the Wong-Baker FACES, FPS-R, NRS, and VAS.

**Conclusions:**

The Emoji-FPS is reliable and valid compared with traditional pain scales in adult surgery patients.

## Introduction

Pain is a complex and subjective experience that is usually measured by self-reported scales [1]. Commonly used unidimensional scales are the visual analog scale (VAS), numerical rating scale (NRS), verbal rating scale, and face pain scale (FPS) [1]. Each of these scales has its own features and application scenarios [2-5].

With the steady development of telemedicine and mobile health in the past two decades, the medical community as well as pain research are adapting themselves to the increasingly digital world [6,7]. In pain monitoring, electronic data capturing methods have robust reliability and validity compared with traditional paper-based data collection methods [8-10]. Electronic pain measurement was more favorable in terms of compliance and patient satisfaction [8]. In the meantime, emerging pain-related smart device apps were developed to collect and track users’ pain information and even provide interventions [10,11].

Although means of collecting data have been greatly developed, fewer improvements were seen for the tools used for measuring pain. The upgrade from pencil-paper to finger-touchscreen interaction provides us with more possibilities including more accurate and timely data capture, and there is a need for an upgrade of the traditional pain scale so that a new pain scale can optimize the potential of smart devices without losing validity and reliability. Evidence revealed that electronically measured VAS, NRS, and FPS have the same performance as those measured by paper and pen [8]. However, VAS and NRS are less appealing to patients compared with FPS [4]. FPS, compared with other types of pain scales, is preferred by people from all age groups and is especially suitable for children and those with cognitive impairment [12,13]. Widely applied FPSs are the Wong-Baker FACES pain rating scale (Wong-Baker FACES) and the faces pain scale-revised (FPS-R), but both of them were developed years ago, and they were designed in monotonic color, which might not be as appealing as colorful faces on digital devices [14,15]. Additionally, traditional FPS might require approval before use and even potential royal fees for commercial applications. As images, it is difficult to guarantee that these traditional scales can fit properly on web pages or mobile apps without being distorted due to a variation in screen sizes and devices. One advantage of using faces to measure pain is that it is more self-explanatory than other types of scales. Using face images that are already popular with users might be more efficient to apply. As pictographs that are typically presented in a colorful form and used inline in text, emoji conform to the Unicode standard and are characterized by standardization and universality. Compared with images, it is flexible to adapt Emoji-FPS to mobile devices without additional image download as emoji have become a preloaded digital set of images that can work across platforms [6]. In addition, emoji are open-source, and they can be easily obtained without approval from anyone.

Digital device users are not unfamiliar with emoji faces such as 
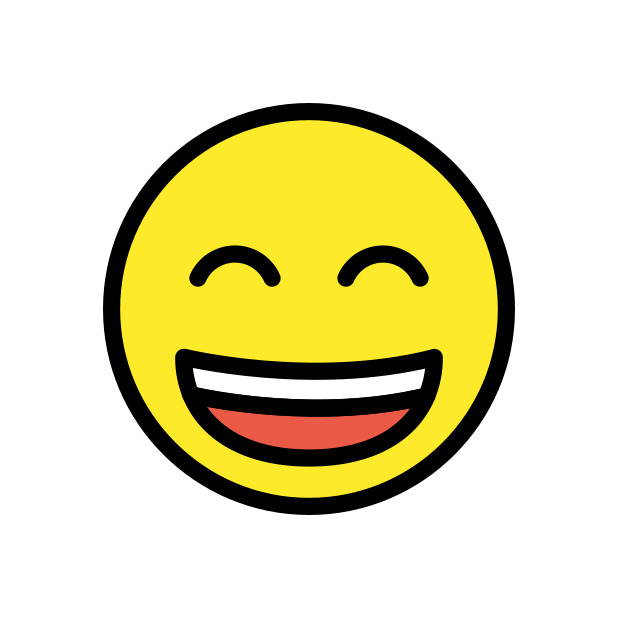
, 
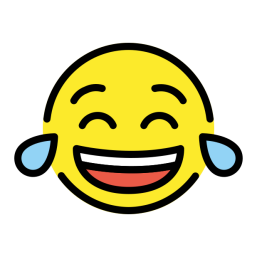
, and 
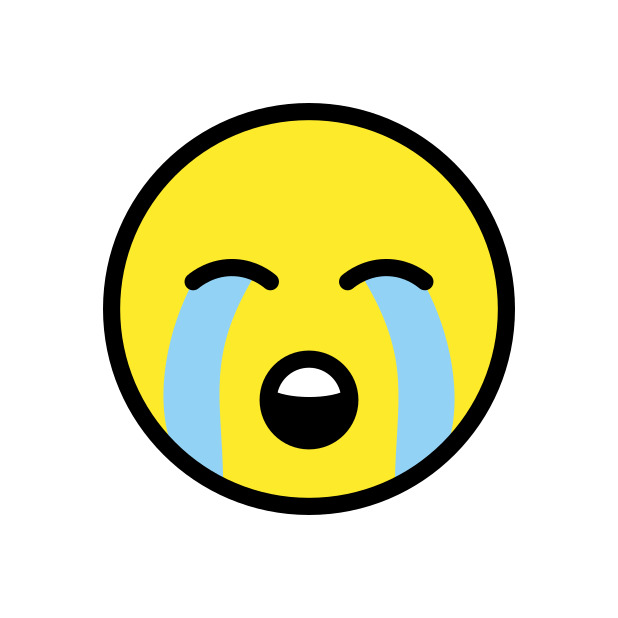
 [6,16]. Until September 2021, a total of 3633 emoji had been created. Among all those emoji, at least 126 face-related emoji can be regarded as potential candidate faces to construct a pain scale. Face pain scales made of emoji faces might be more generalizable to more populations because emoji are not specific to a certain group of people. A certain sequence of emoji faces that can best measure levels of pain needs to be determined before they are applied.

An emoji faces pain scale (Emoji-FPS) should possess consistent reliability and validity compared with classical pain scales. Validation of its usefulness in adults without cognitive impairment first can provide evidence for its further use in other special populations. Patients who underwent perianal surgery are suitable for initial validation of the Emoji-FPS because those patients are usually characterized by mild to moderate preoperative pain and intense postoperative pain, which provides a good opportunity to test the scale’s discriminative validity [17]. In addition, they are also capable of using mobile devices before and after surgery themselves to answer questions.

This study aimed to (1) develop a unidimensional pain scale Emoji-FPS from the emoji faces using a modified Delphi technique; (2) test reliability and validity of the Emoji-FPS in patients scheduled to receive perianal surgery; and (3) evaluate patients’ reference over the VAS, NRS, Wong-Baker FACES, FPS-R, and Emoji-FPS.

## Methods

### Development of Emoji-FPS

#### The Modified Delphi Technique

A modified Delphi technique was used to obtain experts’ consensus on which emoji sequence can best represent different pain levels. Delphi is a method for structuring a group communication process so that the process is effective in allowing a group of individuals, as a whole, to deal with a complex problem [18,19]. The Delphi technique is an iterative process characterized by collecting controlled feedback from experts and requiring some degree of anonymity for the individual responses [20]. The expert panel evaluates and re-evaluates potential items until consensus is met after rounds of sequential surveys [18]. A “modified” Delphi technique was used in this study since the initial emoji sequences for the first round were determined based on literature review and expert interviews [21]. Instead of generating multiple items, only 1 item (1 sequence of emoji) was determined in this study. Reporting of the results of the Delphi survey followed the CHERRIES (Checklist for Reporting Results of Internet E-Surveys) [22].

#### Participants (Delphi Panel)

The expert panel consists of health workers with at least 1 year of working experience in the field of anesthesiology or pain medicine. Both clinicians and nurses were included. Panelists were mainly recruited from the anesthesiology department of Shuguang Hospital affiliated with Shanghai University of Traditional Chinese Medicine. The snowballing technique was applied when panelists’ colleagues from other institutions had interest in participating in this study. According to the rule of thumb, a sample size of 15-30 participants was generally recommended for a homogeneous survey (eg, overlapping specialties exist) [21]. To accommodate possible attrition of panelists from round 1 to round 2, a sample size of 40 was finally chosen.

#### Construction of Initial Emoji Sequences

Since it usually takes months before the latest version of emoji is adapted to major platforms, Emoji version 13.1 (Unicode Consortium) was used in this study [16]. As of August 2021, support for Emoji 13.1 is available on iOS 14.5 (Apple Inc) and various Google platforms. The search for potential emoji faces was confined to the smileys and emotion emoji category. A total of 91 emoji in the following types were selected as candidates: face-smiling, face-affection, face-tongue, face-hand, face-neutral-skeptical, face-sleepy, face-unwell, face-hat, face-glasses, and face-negative. After a literature review, it was found that both widely used FPS—Wong-Baker FACES and FPS-R—choose 6 faces as anchors. It is more practical to transform a scale with 6 levels to either 0-1-2-3-4-5 or 0-2-4-6-8-10; therefore, the 0-5 or the 0-10 metric can be applied. Therefore, our proposed Emoji-FPS also used the 6-level scale scheme.

Both Wong-Baker FACES and FPS-R are well-developed and validated scales over the years, so we aimed to find a sequence of emoji that also possess some properties (curve of eyebrow, shape of eye, curve of mouth, and tears) of these existing face scales so that this sequence of emoji could be more likely to be valid for measuring pain. After discussions of 3 experts (2 experienced in pain and anesthesiology and 1 experienced in public health), a total of 10 sequences were initially constructed for the first round of the survey.

#### Delphi Survey Procedure

The surveys were conducted on the internet from July 2021 to September 2021. Each Delphi survey questionnaire consisted of mandatory instructions for panelists. The panelists were instructed to rate each candidate emoji sequence on a Likert scale from 1 (unsuitable) to 2 (slightly suitable) to 3 (moderately suitable) to 4 (suitable) to 5 (very suitable) based on their feelings about to what extent these emoji sequences can represent the pain levels from “no hurt” to “hurts most.” All panelists’ ratings were equally weighted. Since the appearance of emoji may be different on different devices and platforms, candidate sequences of Emoji-FPS were demonstrated using emoji images from iOS, Android, Microsoft, and OpenMoji versions separately in both rounds of Delphi surveys (see Multimedia Appendix 1). In both rounds, the panelists were instructed to rate each emoji sequence 4 times, once with each version of emoji. OpenMoji was included since it is an open-source project and it is free to share and adapt without restrictive usage rights [23]. Responses of panelists were kept anonymous.

##### Round 1

The link to the web-based survey was officially distributed to 40 potential participants. The panelists were allowed to select emoji beyond those used in the initial 10 sequences if they considered none of the emoji to represent certain levels of pain, and a total of 91 emoji faces are provided in Multimedia Appendix 1. This round was designed to find possible emoji suitable for each level of pain. Based on the results of this round, emoji sequences may be reformed in the next round. The panelists were asked to complete the survey within 3 days, and messages were sent to remind those who had no response after 5 days.

##### Round 2

In this round, invitations of surveys were sent to those who responded in round 1. The survey was in the same form as the previous round. However, fewer emoji sequences were provided, and the number of sequences was subject to the variation in ratings from the previous round. After this round, the final sequence of emoji was determined.

### Validation of Emoji-FPS

#### Participants

Patients scheduled to receive perianal surgery were identified and recruited from the anorectal surgery department of Shuguang Hospital affiliated to Shanghai University of Traditional Chinese Medicine. Inclusion criteria were 18-65 years old and preoperative NRS ≤3. Those with a preoperative mini-mental state examination score <26 or with perianal abscess were excluded.

#### Sample Size

To test consistency between types of Emoji-FPS, pairwise comparisons were made between groups. Assuming the Pearson correlation coefficient was 0.8 (0.6 under the null hypothesis), a sample size of 51 was required to achieve 0.8 power with a significance level of 0.05. Thus, a total of 255 individuals would be needed for the 5 groups. This sample size would also provide enough power for other tests in this study. Considering the dropping out of patients, a final sample size of 300 was determined.

#### Scales

In addition to the Emoji-FPS, a total of 4 well-validated pain scales (NRS, VAS, Wong-Baker FACES, and FPS-R) were selected as references. The 4 versions of Emoji-FPS (iOS, Android, Microsoft platforms, and OpenMoji) were provided (Table S5 in Multimedia Appendix 2).

#### Procedure and Data Collection

Participants were asked to complete the 5 types of pain scales (NRS, VAS, Wong-Baker FACES, FPS-R, and Emoji-FPS) at 5 time points: before surgery (T1), wake up after surgery (T2), 4 hours after surgery (T3), the second day after surgery (T4), and 15 minutes after T4 (T5). Participants’ demographics and surgery information were also collected. To attenuate the influence of the display sequence of the 5 pain scales on the rating result, a total of 5 groups (A, B, C, D, and E) with different sequences of pain scales were designed using a Latin square matrix to ensure that an equal number of participants would rate the 5 scales with a different display sequence (S1, S2, S3, S4, and S5) at each time (see Supplemental Methods section in Multimedia Appendix 2 for details). In addition, 4 versions of the Emoji-FPS were evenly allocated into 4 groups (A, B, C, and D) so that an equal number of participants would rate different versions of the Emoji-FPS at the same time and each participant would rate different versions of the Emoji-FPS at different time except for T4. Participants rated all 4 versions of the Emoji-FPS simultaneously at T4 to evaluate their agreement. After informed consent was obtained, participants were randomly allocated into 1 of the 5 groups using block randomization with a block size of 5. Each participant was provided with a mobile device (eg, iPad) to complete our web-based questionnaire during the study period. At T1, patients completed questionnaires under instruction. Patients can select faces best representing their feeling of pain by taping the radio button with the corresponding scores (0, 2, 4, 6, 8, and 10) displayed below them on the screen, as Wong-Baker FACES and FPS-R did. For NRS, an NRS scale was displayed on screen, and patients could select the corresponding number from a dropdown list. To ensure that different versions of emoji can display well, images of emoji were used. For the remaining follow-ups, patients were reminded of completing questionnaires themselves.

### Ethics Approval

This study was approved by the Chinese Ethics Committee of Registering Clinical Trials (reference number ChiECRCT20210462). Informed consent was obtained from Delphi panelists and patients in the validation step. All study data were deidentified and encrypted when transferred or preserved. Compensation was not provided for participants.

### Statistical Analysis

Descriptive analyses were performed. Spearman rank correlation coefficients (ρ) were calculated to measure the correlation between scales. A paired sample *t* test was used to compare patients’ Emoji-FPS results before and after surgery. The weighted κ coefficient was applied to measure pairwise consistency between versions of the Emoji-FPS. The significance level was .05. SPSS 27.0 (IBM Corp) and SAS 9.4 (SAS Institute Inc) were used for data analysis.

## Results

### Development of Emoji-FPS (Delphi)

Of the 40 invited panel members, all consented and responded to round 1. A total of 36 panelists responded to round 2 with a response rate of 90% (see Multimedia Appendix 2 for a detailed description of the characteristics of panelists and survey results). After 2 rounds of surveys, out of 91 candidate emoji faces, an emoji sequence of 
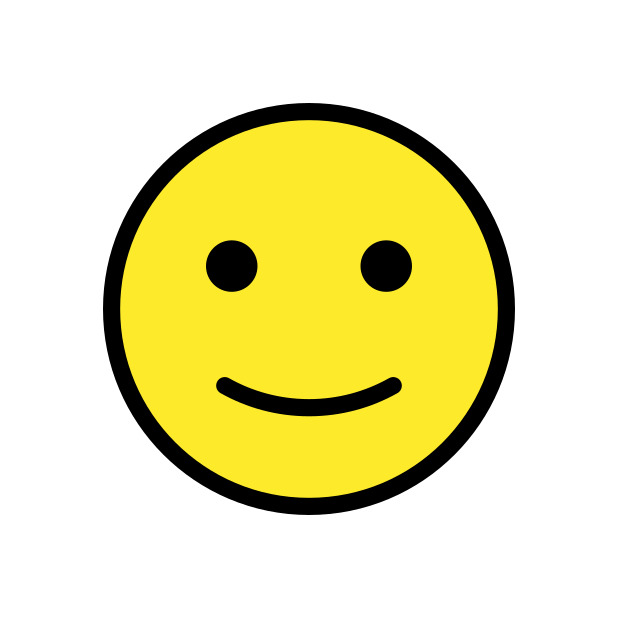
, 
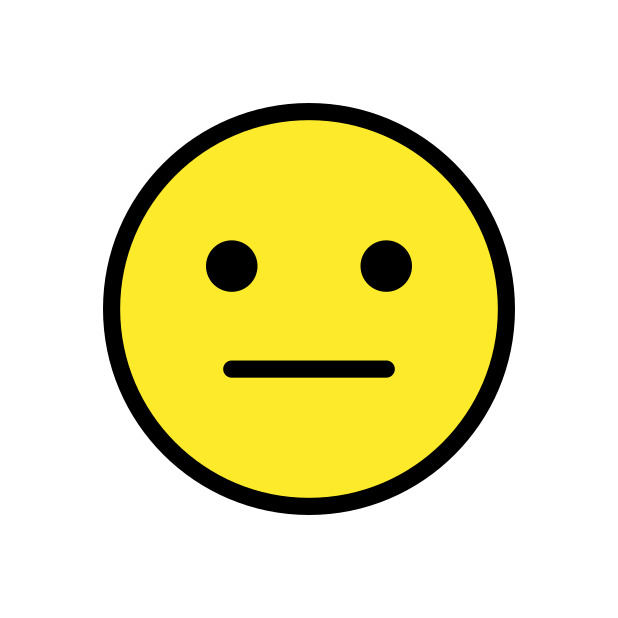
, 
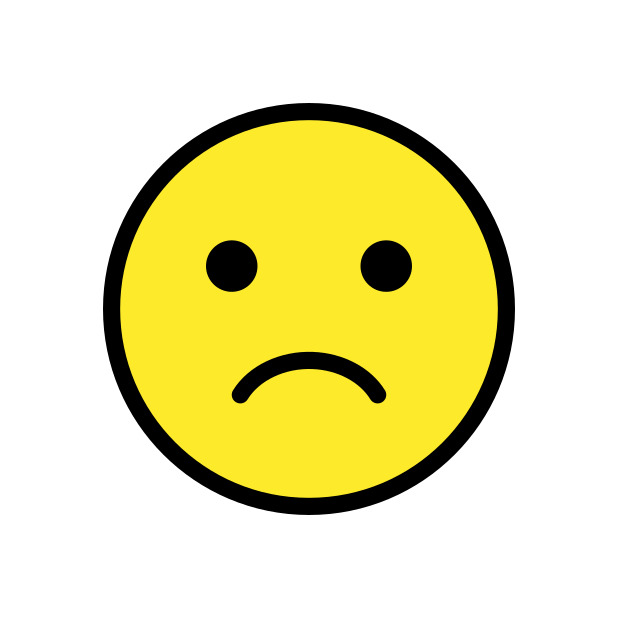
, 
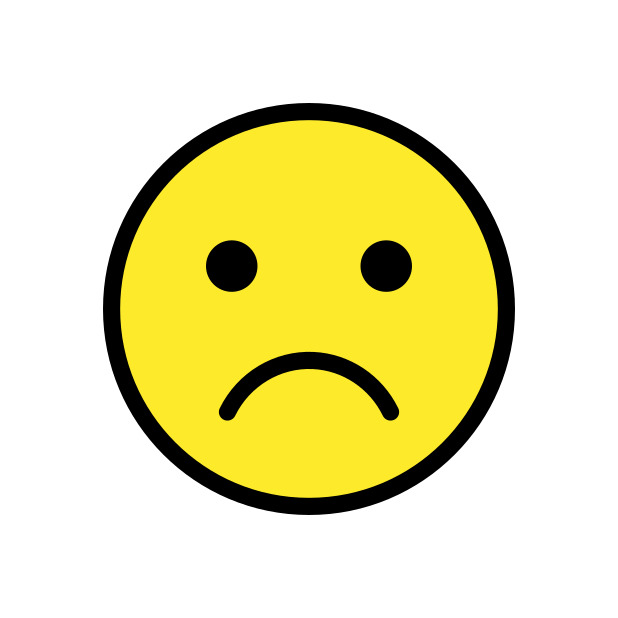
, 
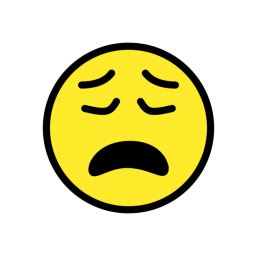
, and 
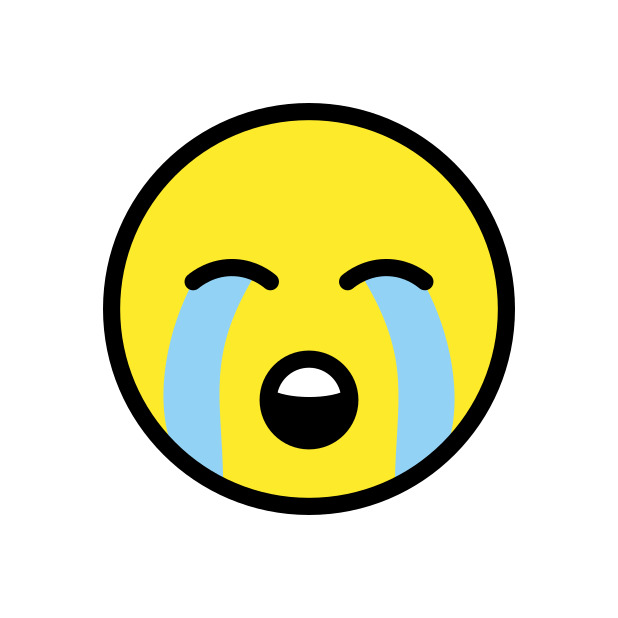
 was finally determined to represent 6 pain levels from “no hurt” to “hurts worst” (Table 1).

**Table 1:** Developed emoji faces pain scale.

	Levels					
	0	2	4	6	8	10
Description	No hurt	Hurts little bit	Hurts little more	Hurts even more	Hurts whole lot	Hurts worst
CLDR^a^ short name	Slightly smiling face	Neutral face	Slightly frowning face	Frowning face	Weary face	Loudly crying face
Code points	U+1F642	U+1F610	U+1F641	U+2639	U+1F629	U+1F62D
OpenMoji^b^	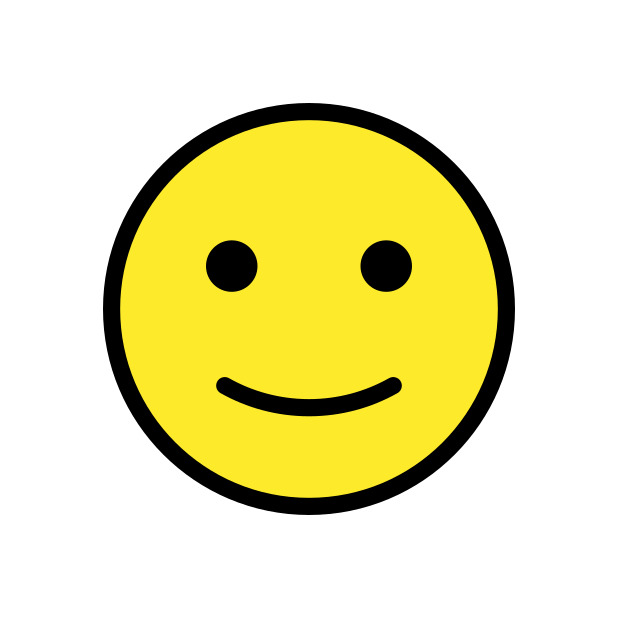	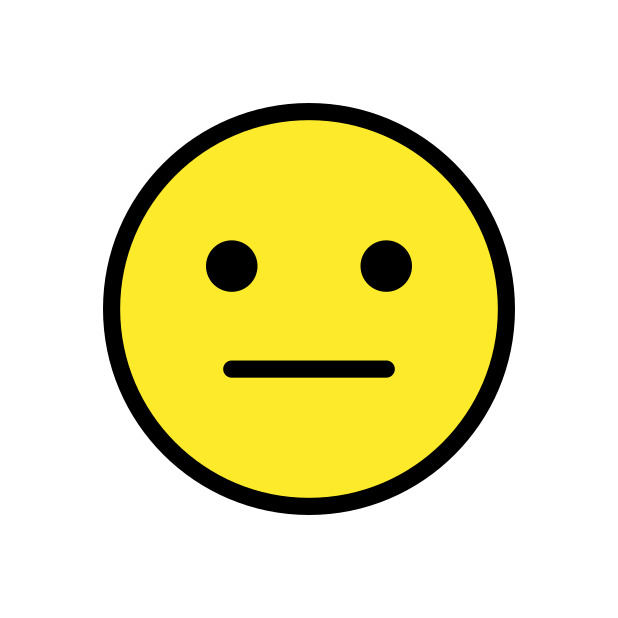	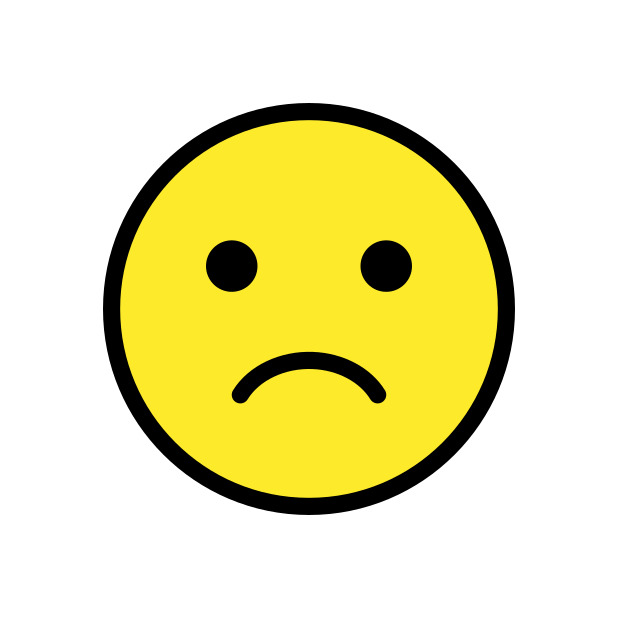	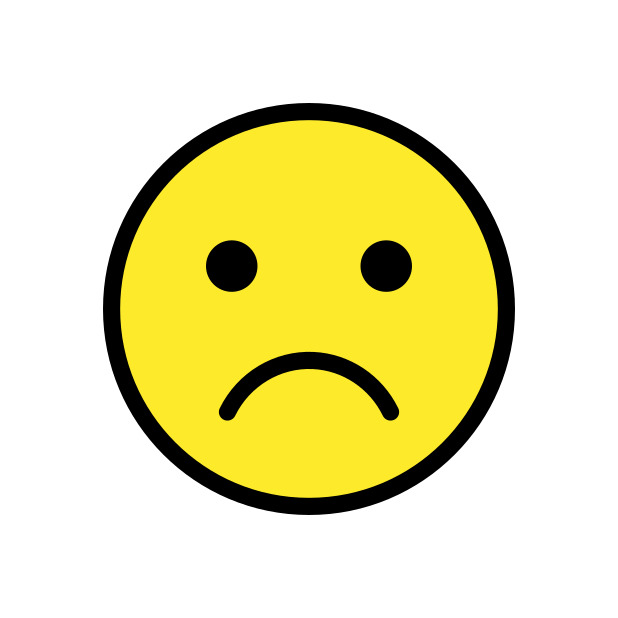	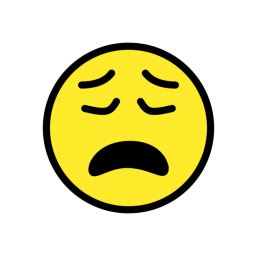	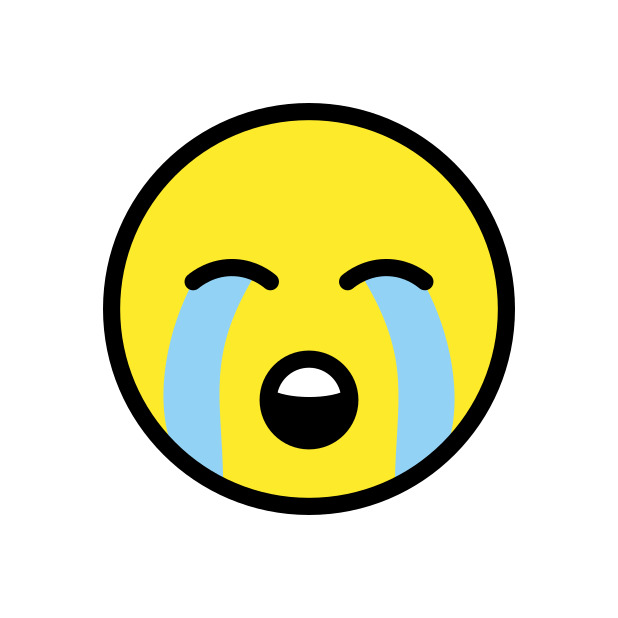

^a^CLDR: The Common Locale Data Repository Project of the Unicode Consortium.

^b^OpenMoji is an open-source project and independent emoji system.

### Validation of Emoji-FPS

#### Patient Characteristics

During November 2021 and January 2022, a total of 352 patients were assessed for eligibility, of whom 300 were randomly allocated to 1 of the 5 groups, and 1 was excluded due to a preoperative NRS score greater than 3. Finally, 299 patients were included in the data analysis (Figure S1 in Multimedia Appendix 2 for flowchart). Study participants had a mean age of 38.5 (SD 10.5) years, and 106 (35.5%) were women (see Table S6 in Multimedia Appendix 2 for details). A total of 68.6% (n=205) of patients had a college or above educational level. Overall, 201 (67.2%) patients received hemorrhoidectomy and 98 (32.8%) received anal fistula resection. The mean surgery duration was 25.9 (SD 11.1) minutes. The distribution of Emoji-FPS scores covered 0 to 10 over T1 to T5 (see Figure S2 in Multimedia Appendix 2).

#### Validity

##### Concurrent Validity

Generally, Emoji-FPS was highly correlated with 4 reference scales with ρ ranging from 0.91 to 0.95 (Table 2). The associations between the Emoji-FPS and 4 reference scales at each time point were satisfactory, with ρ values ranging from 0.89 to 0.96. Each version of the Emoji-FPS also revealed a sound correlation with 4 reference scales (range of ρ=0.67-0.98). The agreements between different types of Emoji-FPS were high (range of weighted κ coefficients=0.96-0.97; see Table S7 in Multimedia Appendix 2).

**Table 2:** Results of concurrent validity test.

Reference scale and time point			Types of Emoji-FPS^a^, ρ								
			iOS		Android		Microsoft		OpenMoji		Combined
**NRS^b^**											
	T1	0.84		0.88		0.67		0.91		0.82	
	T2	0.93		0.94		0.91		0.89		0.92	
	T3	0.91		0.91		0.85		0.93		0.89	
	T4	0.92		0.89		0.95		0.90		0.90	
	Total	0.93		0.94		0.89		0.93		0.92	
**VAS^c^**											
	T1	0.74		0.85		0.75		0.81		0.81	
	T2	0.93		0.91		0.91		0.88		0.91	
	T3	0.93		0.86		0.88		0.92		0.89	
	T4	0.88		0.88		0.90		0.90		0.90	
	Total	0.92		0.92		0.89		0.92		0.91	
**WB FACES^d^**											
	T1	0.96		0.95		0.88		0.72		0.88	
	T2	0.94		0.94		0.91		0.91		0.93	
	T3	0.97		0.91		0.85		0.98		0.92	
	T4	0.93		0.98		0.95		0.94		0.93	
	Total	0.96		0.96		0.92		0.94		0.94	
**FPS-R^e^**											
	T1	0.88		0.91		0.93		0.96		0.91	
	T2	0.93		0.90		0.91		0.95		0.92	
	T3	0.91		0.93		0.93		0.95		0.93	
	T4	0.98		0.98		0.98		0.91		0.96	
	Total	0.94		0.95		0.95		0.95		0.95	

^a^Emoji-FPS: emoji faces pain scale.

^b^NRS: numerical rating scale.

^c^VAS: visual analog scale.

^d^WB FACES: Wong-Baker FACES.

^e^FPS-R: faces pain scale-revised.

##### Discriminant Validity

For all participants, their postsurgery mean Emoji-FPS score was 1.4 points (95% CI 1.3-1.6; *P*<.001) higher than the presurgery results, indicating a sound discriminant validity (Table 3).

**Table 3:** Results of the discriminant validity test.

Time point	Types of Emoji-FPS^a^				
	iOS	Android	Microsoft	OpenMoji	Combined
T1, mean (SD)	1.4 (0.7)	1.3 (0.5)	1.5 (0.7)	1.4 (0.6)	1.4 (0.7)
T4, mean (SD)	2.9 (1.2)	2.5 (1.0)	2.9 (1.3)	2.6 (1.2)	2.8 (1.2)
Difference (95% CI)	1.6 (1.3-1.9)	1.2 (0.9-1.1)	1.4 (1.1-1.8)	1.3 (1.0-1.6)	1.4 (1.3-1.6)
*P* value^b^	<.001	<.001	<.001	<.001	<.001

^a^Emoji-FPS: emoji faces pain scale.

^b^Calculated using paired *t* test.

#### Reliability

Good test-retest reliability was supported by the high correlation between the Emoji-FPS results at T4 and T5 (range of ρ=0.88-0.98; Table 4).

**Table 4:** Results of the test-retest reliability test.

Time point	Types of Emoji-FPS^a^				
	iOS	Android	Microsoft	OpenMoji	Combined
T4, mean (SD)	2.9 (1.2)	2.5 (1.0)	2.9 (1.3)	2.6 (1.2)	2.8 (1.2)
T5, mean (SD)	2.9 (1.2)	2.5 (1.1)	2.9 (1.2)	2.7 (1.2)	2.7 (1.1)
ρ^b^	0.88	0.90	0.98	0.88	0.91

^a^Emoji-FPS: emoji faces pain scale.

^b^Spearman correlation coefficient for results between T4 and T5.

#### Scale Preference

**Figure 1:**
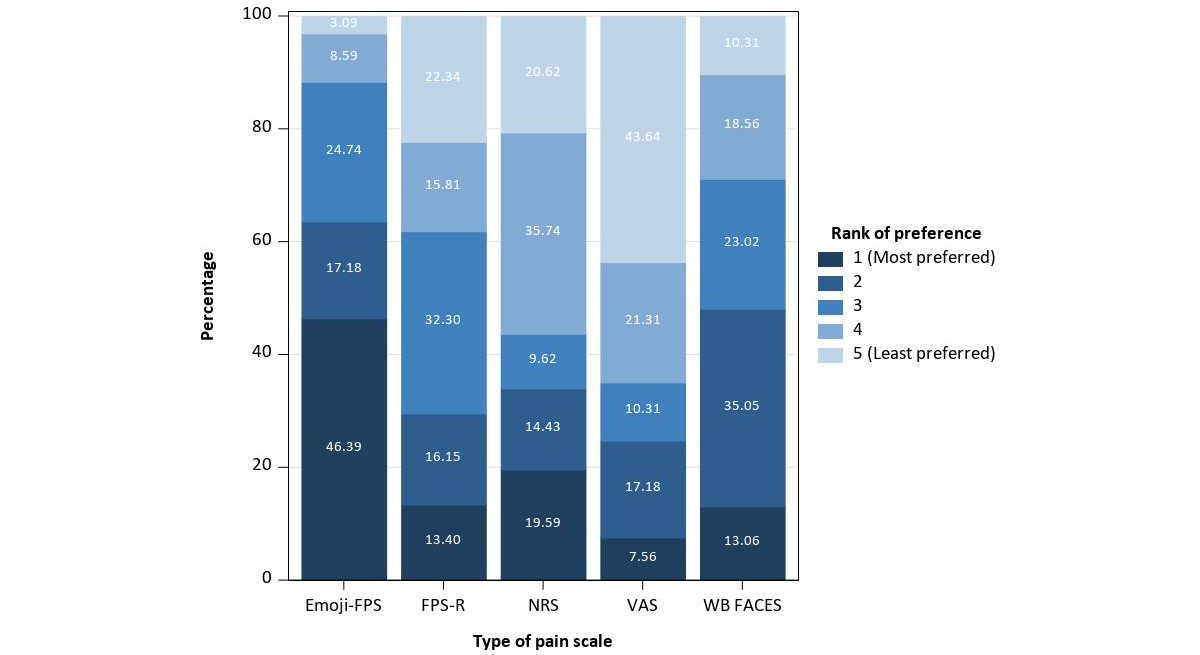
Distribution of patients’ rank of preference of 5 pain scales. Emoji-FPS: emoji faces pain scale; FPS-R: faces pain scale-revised; NRS: numerical rating scale; VAS: visual analog scale; WB FACES: Wong-Baker FACES.

Emoji-FPS was mostly welcomed by participants (mean 2.0, SD 1.2), followed by Wong-Baker FACES (mean 2.8, SD 1.2), FPS-R (mean 3.2, SD 1.3), NRS (mean 3.2, SD 1.4), and VAS (mean 3.8, SD 1.4). See Figure 1 for a detailed distribution.

## Discussion

### Principal Findings

In this study, a 6-level Emoji-FPS (
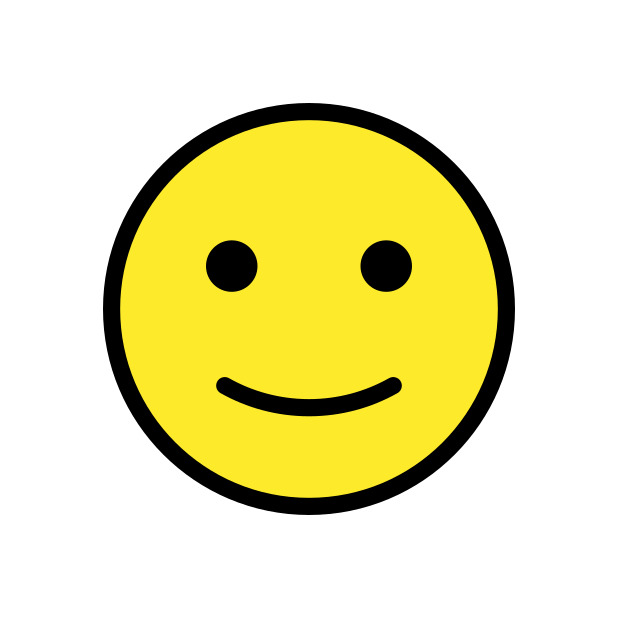
, 
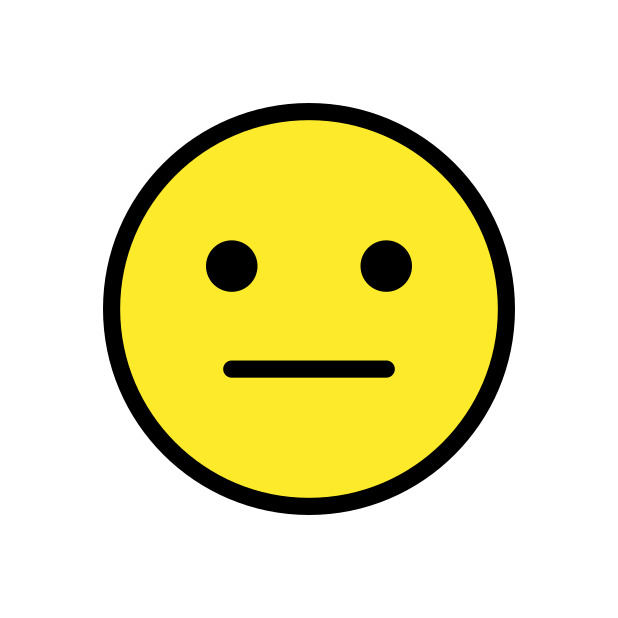
, 
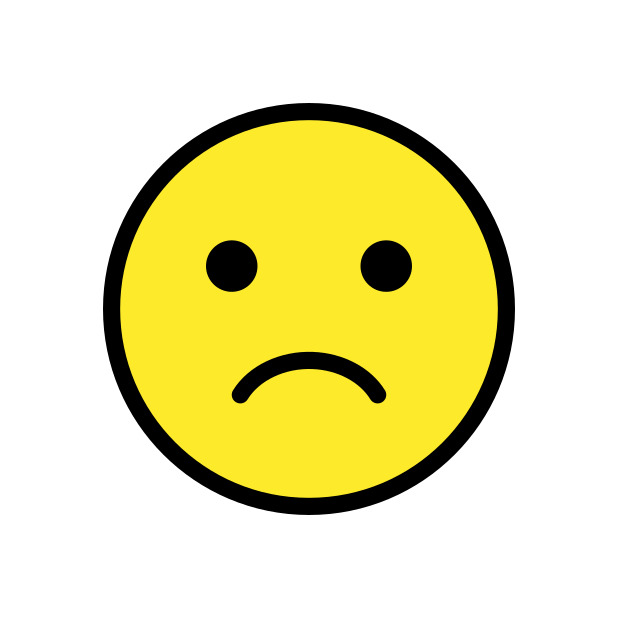
, 
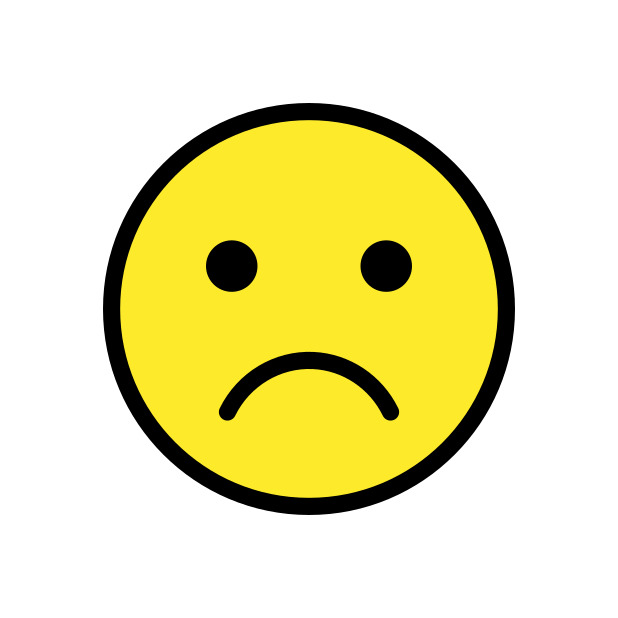
, 
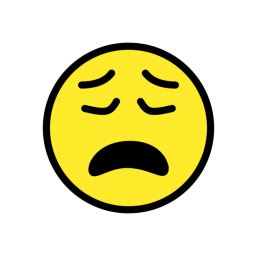
, and 
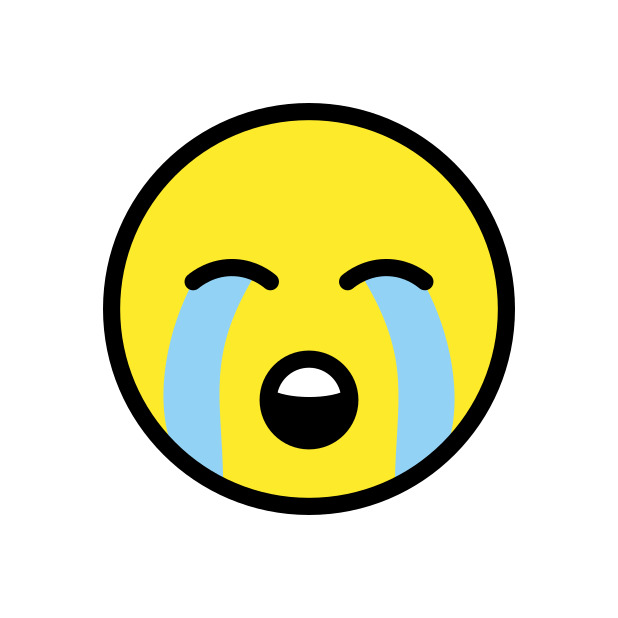
) was developed. Satisfactory validity and reliability of the Emoji-FPS were confirmed in patients who underwent perianal surgery.

The potential use of emoji in medical research has been discussed previously, although its application is still limited [6,7]. Emoji were considered to be used as an indicator to evaluate patient-reported outcomes in breast cancer treatment due to its friendliness on social networking services and electronic devices [24]. Kiliç et al [25] used emoji as anchors to replace common numerical anchors in the Likert-type Psychological Well-Being Scale. They used 
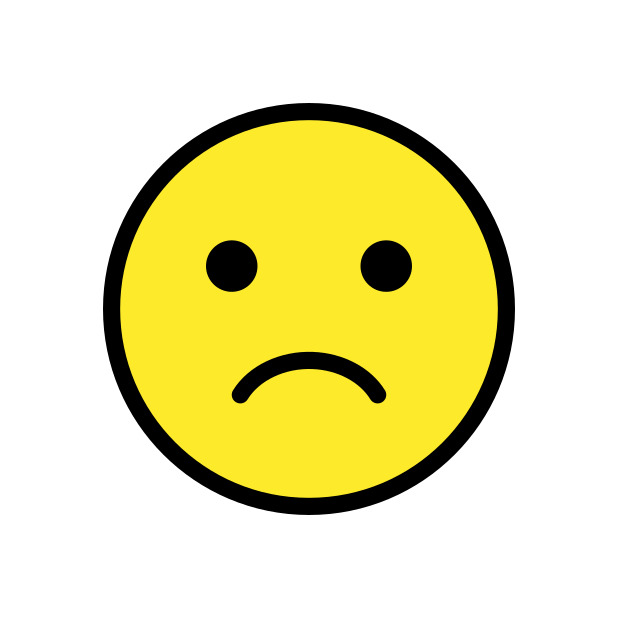
, 
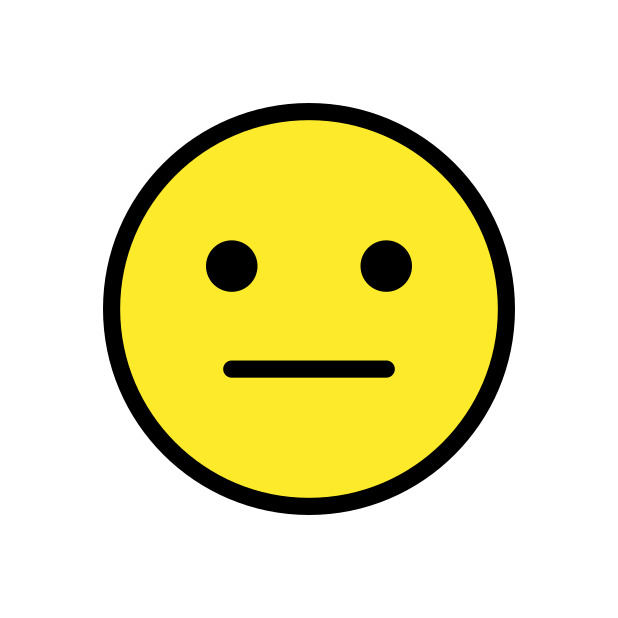
, and 
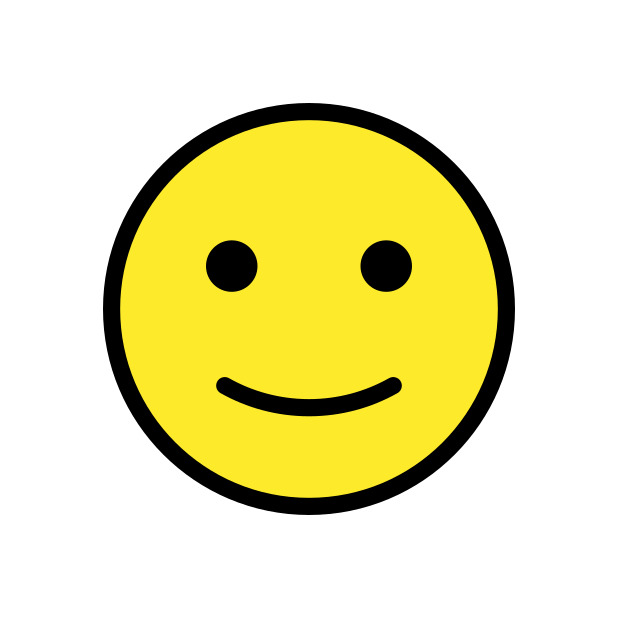
 to represent a 3-point scale (disagree, neutral, and agree); 
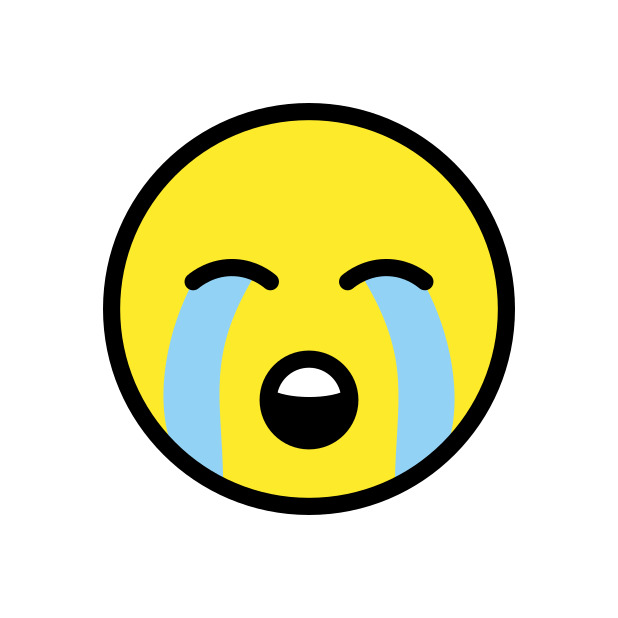
, 
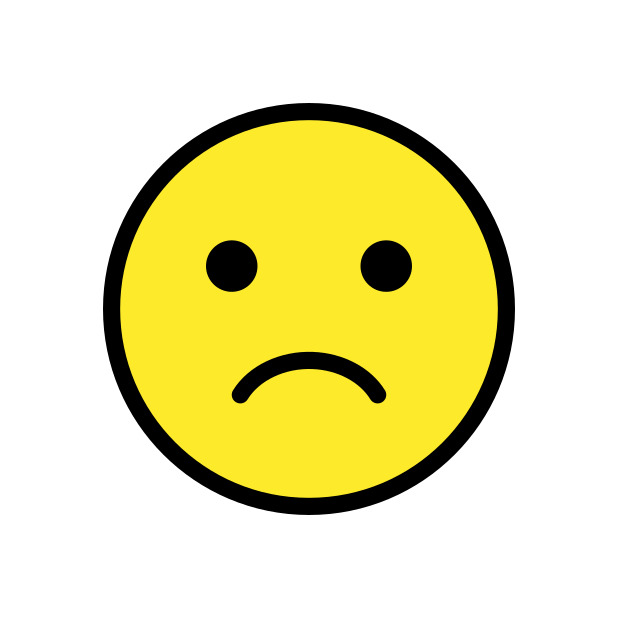
, 
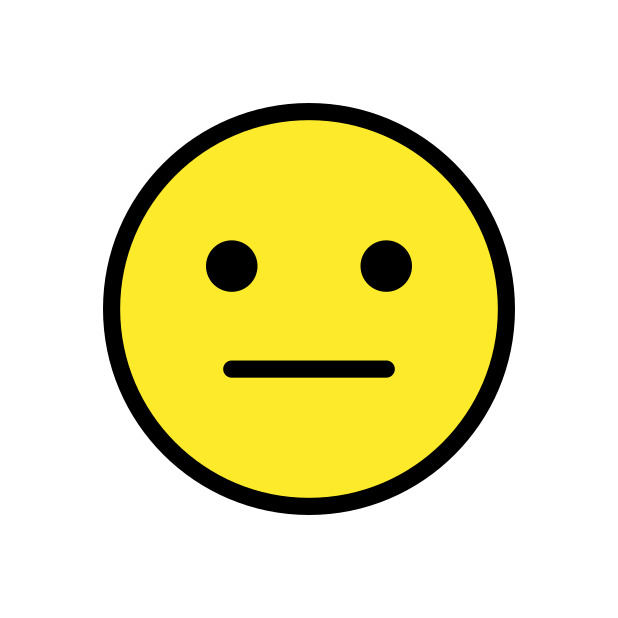
, 
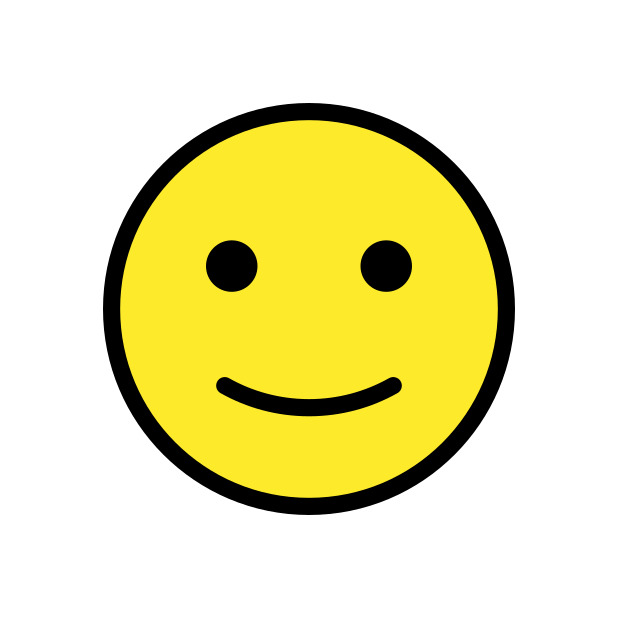
, and 
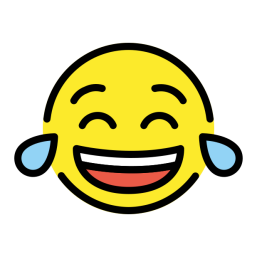
 to represent a 5-point scale (from strongly disagree to strongly agree), and 
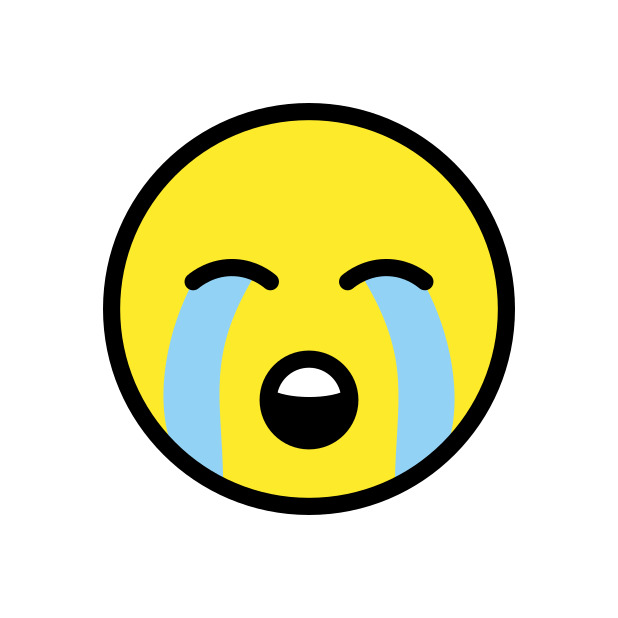
, 
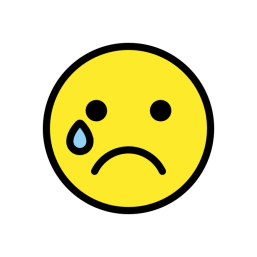
, 
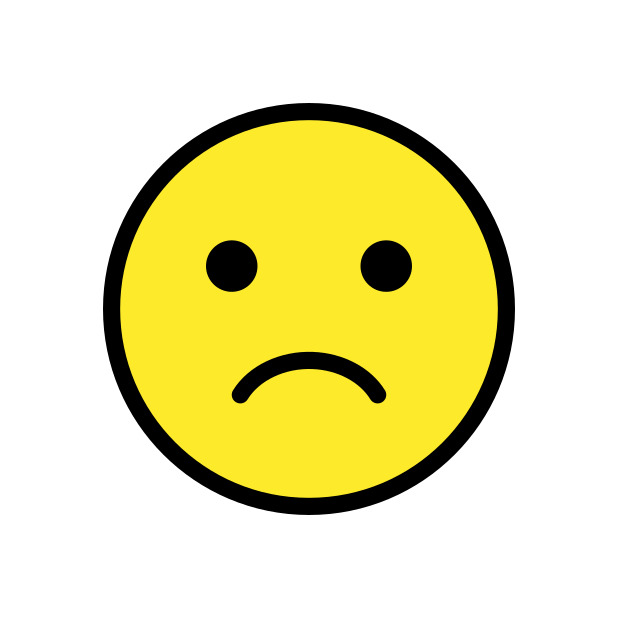
, 
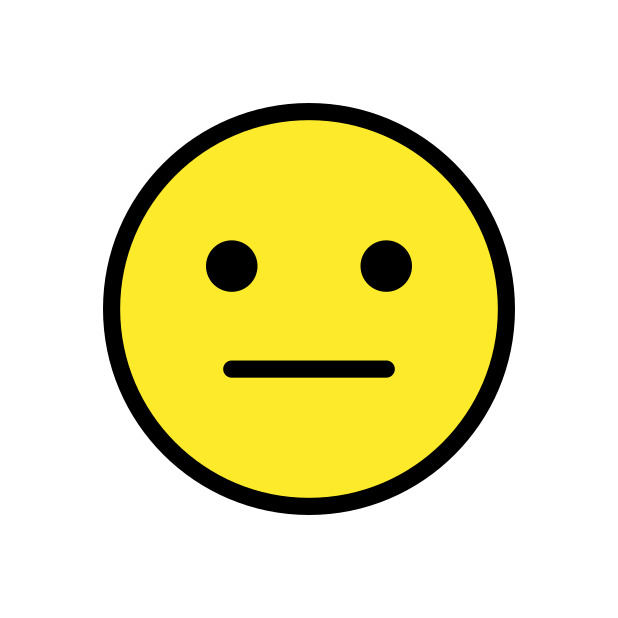
, 
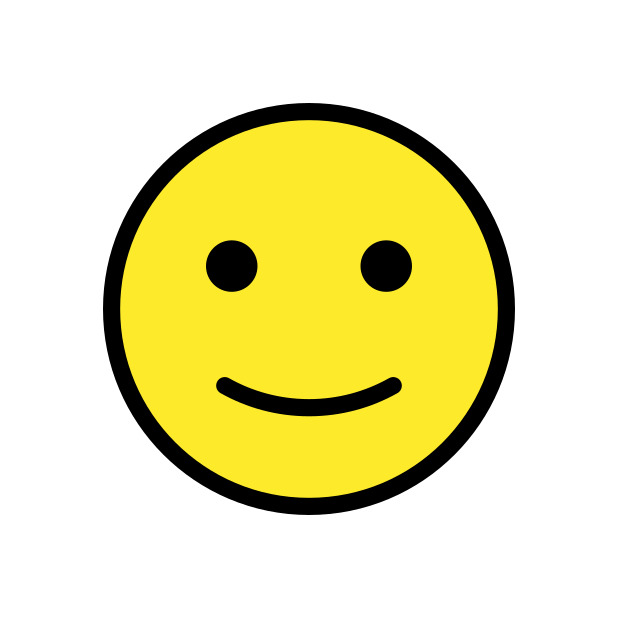
, 
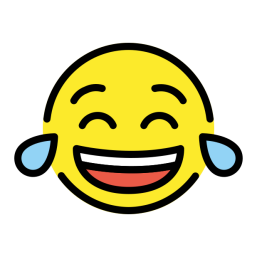
, and 
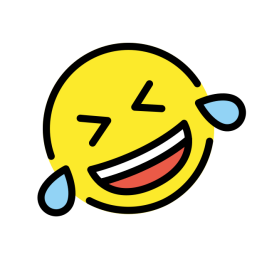
 to represent a 7-point scale (from strongly disagree to absolutely agree). However, as O’Reilly-Shah et al [7] commented, “two interrelated problems are the durability of an Emoji’s meaning over time and the potential variability in its meaning in different cultural and linguistic contexts.” For example, “face with tears of joy” (
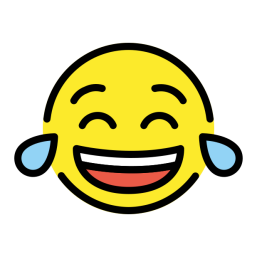
) can be used to convey the feeling of “laughing so hard I’m crying,” and it is significantly different from its named intent. This is one of the reasons why we chose to use a modified Delphi technique to determine the Emoji-FPS. Our developed Emoji-FPS was reached upon collated opinions from a group of people to avoid the subjective choice of emoji. He et al [26] also developed an Emoji-FPS with a sequence of 
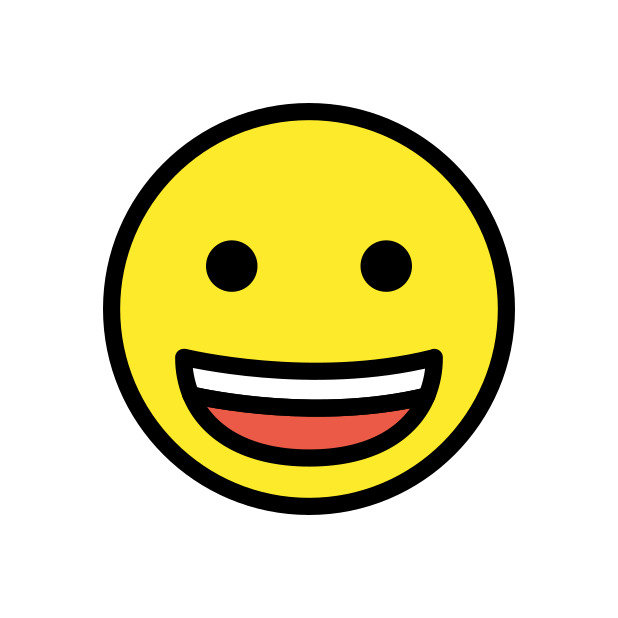
, 
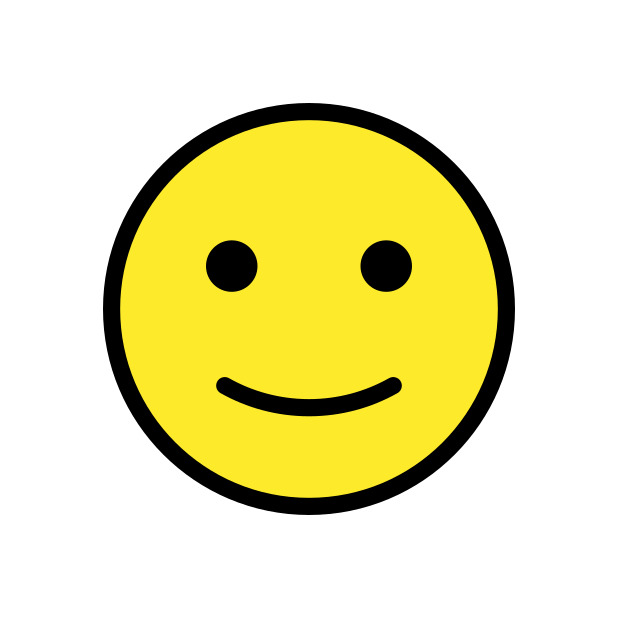
, 
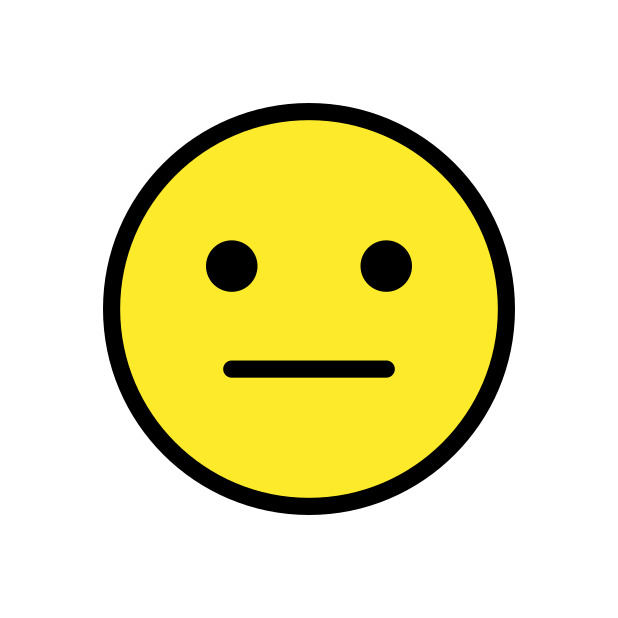
, 
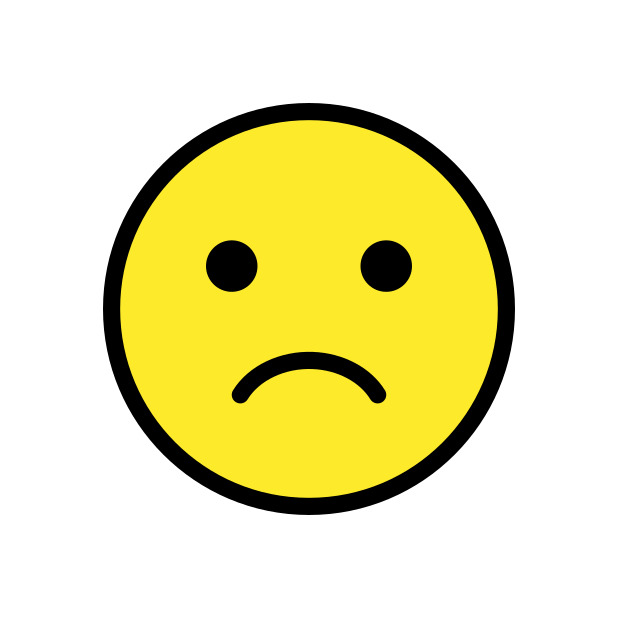
, 
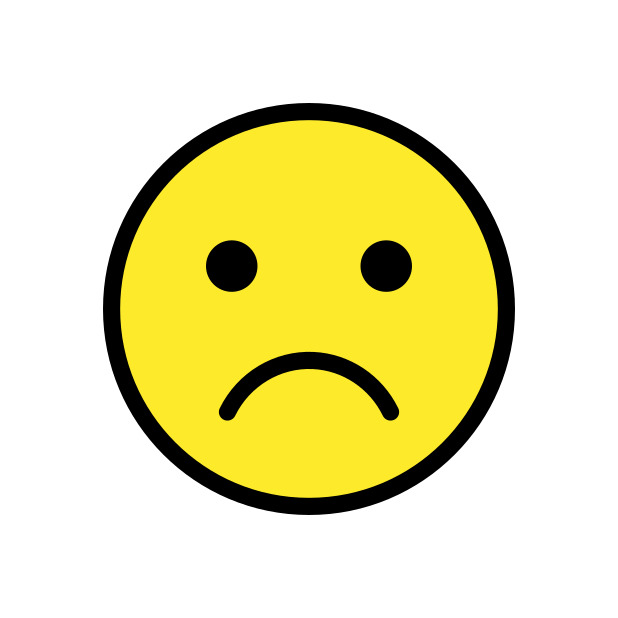
, and 
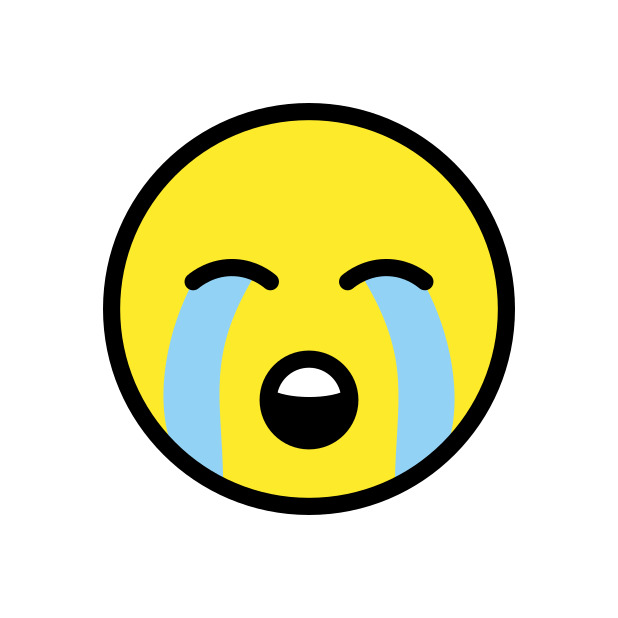
. A high level of agreement was observed between their Emoji-FPS and VAS after validation in 109 patients. Compared with their proposed Emoji-FPS, our proposed Emoji-FPS does not contain the smiling face 
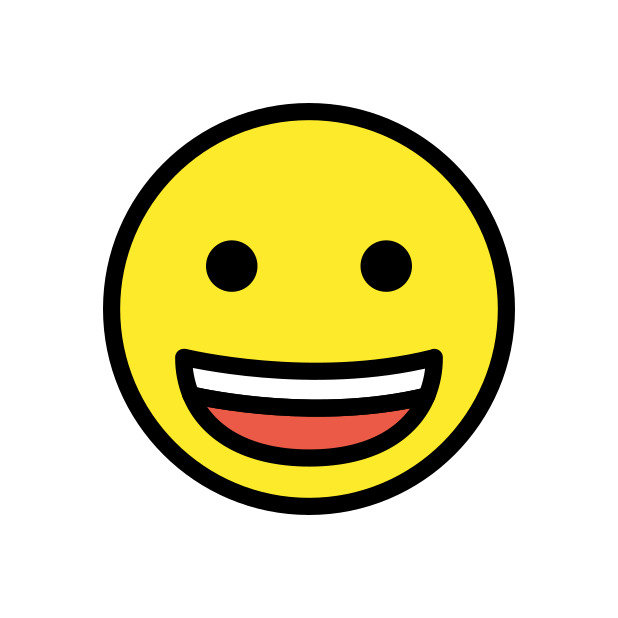
 and includes a painful face 
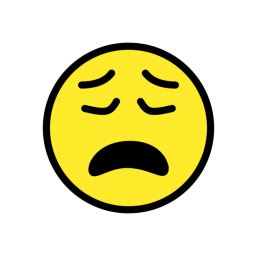
. Since smiles can be reminiscent of happiness, starting with a more neutral face might be more advantageous [27]. Before wide application, more validation studies are needed to reach a consensus.

It is not surprising that the Emoji-FPS was preferred by patients, followed by the Wong-Baker FACES, FPS-R, NRS, and VAS. Colorful and intuitive images could be more attractive to users than monotonic and abstract images, especially on mobile devices. In addition, emoji have fewer details than Wong-Baker FACES and FPS-R. While these details can make faces of different pain levels more distinguishable, excessive details might make users think that these faces no longer look like them, for example, if patients have a different gender, age, or race. Emoji are more concisely designed and are not limited to these specific characteristics, so they have the potential to be applied regardless of age, gender, and race.

Unlike typefaces and fonts of characters appearing exactly the same across platforms, emoji could have different appearances between devices and platforms, although the meanings they convey should be unified. As the Unicode Consortium states, “while the shape of the character can vary significantly, designers should maintain the same core’ shape, based on the shapes used mostly commonly in industry practice” [28]. In this study, excellent agreement was observed between different versions of the Emoji-FPS. It is flexible to adapt Emoji-FPS to mobile devices as emoji has become a preloaded digital set of images that can work across platforms [6]. Moreover, as users of digital devices have become more familiar with them, the Emoji-FPS can be applied across language and communication barriers (eg, patients with dysphasia or patients intubated and unable to communicate verbally). However, as the versions or variations of emoji emerge, the additional evaluation of the reliability and validity of other versions of emoji before application would be useful.

### Limitations

This study has limitations. Only health workers were invited to decide the Emoji-FPS from thousands of emoji, and opinions from patients were not considered. However, the relatively large panel size may accommodate this shortcoming. Our Emoji-FPS was only validated among adult surgery patients in a single center, and generalization of the Emoji-FPS to other populations, such as children, might be limited. Qualitative interviews with children or patients will be planned before further validation. Due to a limitation of the design of the web-based questionnaire, patients needed to select answers to the NRS from a dropdown list, so patients’ experience of using the NRS might be compromised. Their preference for NRS might change if a better interface is provided.

### Conclusions

Our developed Emoji-FPS has been proven to be reliable and valid compared with traditional pain scales on mobile devices among perioperative patients. Further studies are needed to confirm the reliability and validity of the Emoji-FPS in other settings.

## Appendix

Multimedia Appendix 1 Questionnaires of the Delphi survey.

Multimedia Appendix 2 Supplemental results and methods.

## Abbreviations

CHERRIES: Checklist for Reporting Results of Internet E-Surveys
Emoji-FPS: emoji faces pain scale
FPS: face pain scale
FPS-R: faces pain scale-revised
NRS: numerical rating scale
VAS: visual analog scale
Wong-Baker FACES: Wong-Baker FACES pain rating scale

## Data Availability

Research data are available upon reasonable request.
