# Supplementary material for: Development of the Emoji Faces Pain Scale and Its Validation on Mobile Devices in Adult Surgery Patients: Longitudinal Observational Study
Source: J Med Internet Res. 2023 Apr 17;25:e41189. doi: 10.2196/41189 (PMC10152337; doi:10.2196/41189)
Supplement: Multimedia Appendix 1 [file jmir_v25i1e41189_app1.docx]

# Multimedia Appendix 1. Questionnaires of the Delphi survey.

1. Round 1 Survey Questionnaire.

2. Round 2 Survey Questionnaire.

Round 1 Survey Questionnaire (demographics forms omitted).

| Emoji sequences | Levels of pain | | | | | | Please rate each emoji sequence on a Likert scale, from 1 (“unsuitable”) to 2 (“slightly suitable”) to 3 (“moderate suitable”) to 4 (“suitable”) to 5 (“very suitable”) based on your feelings about to what extent the emoji sequence can represent the pain levels from “no hurt” to “hurts most”. | | | | |
| --- | --- | --- | --- | --- | --- | --- | --- | --- | --- | --- | --- |
|  | 0 | 2 | 4 | 6 | 8 | 10 |  |  |  |  |  |
|  | No hurt | Hurts Little Bit | Hurts Little More | Hurts Even More | Hurts Whole Lot | Hurts Worst | unsuitable | slightly suitable | moderate suitable | suitable | very suitable |
| A1 | [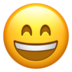](https://emojipedia.org/grinning-face-with-smiling-eyes/) | [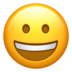](https://emojipedia.org/grinning-face/) | [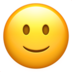](https://emojipedia.org/slightly-smiling-face/) | [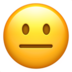](https://emojipedia.org/neutral-face/) | [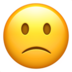](https://emojipedia.org/slightly-frowning-face/) | [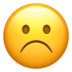](https://emojipedia.org/frowning-face/) |  |  |  |  |  |
| B1 | [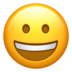](https://emojipedia.org/grinning-face/) | [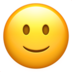](https://emojipedia.org/slightly-smiling-face/) | [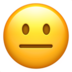](https://emojipedia.org/neutral-face/) | [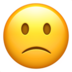](https://emojipedia.org/slightly-frowning-face/) | [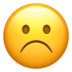](https://emojipedia.org/frowning-face/) | [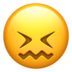](https://emojipedia.org/confounded-face/) |  |  |  |  |  |
| C1 | [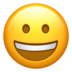](https://emojipedia.org/grinning-face/) | [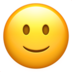](https://emojipedia.org/slightly-smiling-face/) | [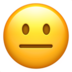](https://emojipedia.org/neutral-face/) | [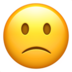](https://emojipedia.org/slightly-frowning-face/) | [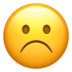](https://emojipedia.org/frowning-face/) | [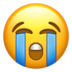](https://emojipedia.org/loudly-crying-face/) |  |  |  |  |  |
| D1 | [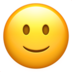](https://emojipedia.org/slightly-smiling-face/) | [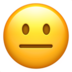](https://emojipedia.org/neutral-face/) | [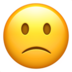](https://emojipedia.org/slightly-frowning-face/) | [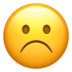](https://emojipedia.org/frowning-face/) | [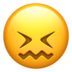](https://emojipedia.org/confounded-face/) | [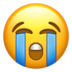](https://emojipedia.org/loudly-crying-face/) |  |  |  |  |  |
| E1 | [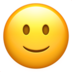](https://emojipedia.org/slightly-smiling-face/) | [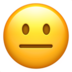](https://emojipedia.org/neutral-face/) | [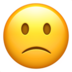](https://emojipedia.org/slightly-frowning-face/) | [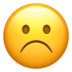](https://emojipedia.org/frowning-face/) | [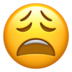](https://emojipedia.org/weary-face/) | [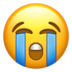](https://emojipedia.org/loudly-crying-face/) |  |  |  |  |  |
| F1 | [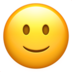](https://emojipedia.org/slightly-smiling-face/) | [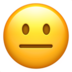](https://emojipedia.org/neutral-face/) | [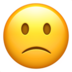](https://emojipedia.org/slightly-frowning-face/) | [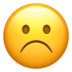](https://emojipedia.org/frowning-face/) | [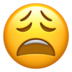](https://emojipedia.org/weary-face/) | [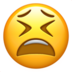](https://emojipedia.org/tired-face/) |  |  |  |  |  |
| G1 | [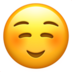](https://emojipedia.org/smiling-face/) | [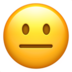](https://emojipedia.org/neutral-face/) | [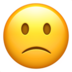](https://emojipedia.org/slightly-frowning-face/) | [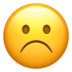](https://emojipedia.org/frowning-face/) | [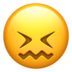](https://emojipedia.org/confounded-face/) | [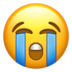](https://emojipedia.org/loudly-crying-face/) |  |  |  |  |  |
| H1 | [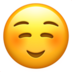](https://emojipedia.org/smiling-face/) | [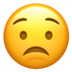](https://emojipedia.org/worried-face/) | [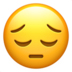](https://emojipedia.org/pensive-face/) | [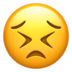](https://emojipedia.org/persevering-face/) | [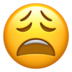](https://emojipedia.org/weary-face/) | [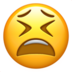](https://emojipedia.org/tired-face/) |  |  |  |  |  |
| I1 | [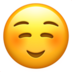](https://emojipedia.org/smiling-face/) | [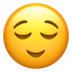](https://emojipedia.org/relieved-face/) | [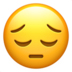](https://emojipedia.org/pensive-face/) | [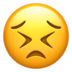](https://emojipedia.org/persevering-face/) | [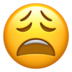](https://emojipedia.org/weary-face/) | [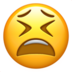](https://emojipedia.org/tired-face/) |  |  |  |  |  |
| J1 | [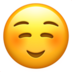](https://emojipedia.org/smiling-face/) | [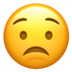](https://emojipedia.org/worried-face/) | [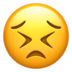](https://emojipedia.org/persevering-face/) | [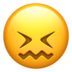](https://emojipedia.org/confounded-face/) | [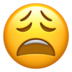](https://emojipedia.org/weary-face/) | [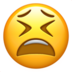](https://emojipedia.org/tired-face/) |  |  |  |  |  |

| Emoji sequences | Levels of pain | | | | | | Please rate each emoji sequence on a Likert scale, from 1 (“unsuitable”) to 2 (“slightly suitable”) to 3 (“moderate suitable”) to 4 (“suitable”) to 5 (“very suitable”) based on your feelings about to what extent the emoji sequence can represent the pain levels from “no hurt” to “hurts most”. | | | | |
| --- | --- | --- | --- | --- | --- | --- | --- | --- | --- | --- | --- |
|  | 0 | 2 | 4 | 6 | 8 | 10 |  |  |  |  |  |
|  | No hurt | Hurts Little Bit | Hurts Little More | Hurts Even More | Hurts Whole Lot | Hurts Worst | unsuitable | slightly suitable | moderate suitable | suitable | very suitable |
| A2 | [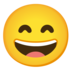](https://emojipedia.org/grinning-face-with-smiling-eyes/) | [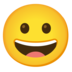](https://emojipedia.org/grinning-face/) | [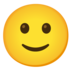](https://emojipedia.org/slightly-smiling-face/) | [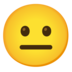](https://emojipedia.org/neutral-face/) | [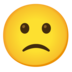](https://emojipedia.org/slightly-frowning-face/) | [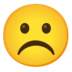](https://emojipedia.org/frowning-face/) |  |  |  |  |  |
| B2 | [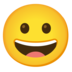](https://emojipedia.org/grinning-face/) | [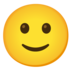](https://emojipedia.org/slightly-smiling-face/) | [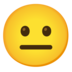](https://emojipedia.org/neutral-face/) | [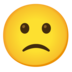](https://emojipedia.org/slightly-frowning-face/) | [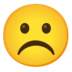](https://emojipedia.org/frowning-face/) | [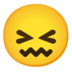](https://emojipedia.org/confounded-face/) |  |  |  |  |  |
| C2 | [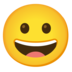](https://emojipedia.org/grinning-face/) | [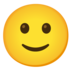](https://emojipedia.org/slightly-smiling-face/) | [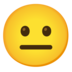](https://emojipedia.org/neutral-face/) | [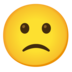](https://emojipedia.org/slightly-frowning-face/) | [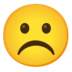](https://emojipedia.org/frowning-face/) | [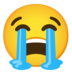](https://emojipedia.org/loudly-crying-face/) |  |  |  |  |  |
| D2 | [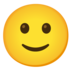](https://emojipedia.org/slightly-smiling-face/) | [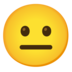](https://emojipedia.org/neutral-face/) | [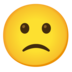](https://emojipedia.org/slightly-frowning-face/) | [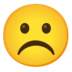](https://emojipedia.org/frowning-face/) | [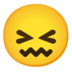](https://emojipedia.org/confounded-face/) | [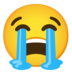](https://emojipedia.org/loudly-crying-face/) |  |  |  |  |  |
| E2 | [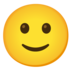](https://emojipedia.org/slightly-smiling-face/) | [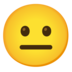](https://emojipedia.org/neutral-face/) | [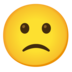](https://emojipedia.org/slightly-frowning-face/) | [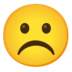](https://emojipedia.org/frowning-face/) | [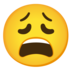](https://emojipedia.org/weary-face/) | [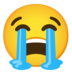](https://emojipedia.org/loudly-crying-face/) |  |  |  |  |  |
| F2 | [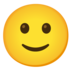](https://emojipedia.org/slightly-smiling-face/) | [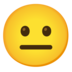](https://emojipedia.org/neutral-face/) | [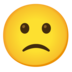](https://emojipedia.org/slightly-frowning-face/) | [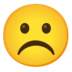](https://emojipedia.org/frowning-face/) | [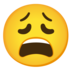](https://emojipedia.org/weary-face/) | [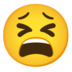](https://emojipedia.org/tired-face/) |  |  |  |  |  |
| G2 | [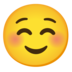](https://emojipedia.org/smiling-face/) | [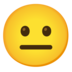](https://emojipedia.org/neutral-face/) | [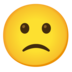](https://emojipedia.org/slightly-frowning-face/) | [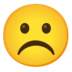](https://emojipedia.org/frowning-face/) |  |  |  |  |  |  |  |
| H2 |  |  |  |  |  |  |  |  |  |  |  |
| I2 |  |  |  |  |  |  |  |  |  |  |  |
| J2 |  |  |  |  |  |  |  |  |  |  |  |

| Emoji sequences | Levels of pain | | | | | | Please rate each emoji sequence on a Likert scale, from 1 (“unsuitable”) to 2 (“slightly suitable”) to 3 (“moderate suitable”) to 4 (“suitable”) to 5 (“very suitable”) based on your feelings about to what extent the emoji sequence can represent the pain levels from “no hurt” to “hurts most”. | | | | |
| --- | --- | --- | --- | --- | --- | --- | --- | --- | --- | --- | --- |
|  | 0 | 2 | 4 | 6 | 8 | 10 |  |  |  |  |  |
|  | No hurt | Hurts Little Bit | Hurts Little More | Hurts Even More | Hurts Whole Lot | Hurts Worst | unsuitable | slightly suitable | moderate suitable | suitable | very suitable |
| A3 |  |  |  |  |  |  |  |  |  |  |  |
| B3 |  |  |  |  |  |  |  |  |  |  |  |
| C3 |  |  |  |  |  |  |  |  |  |  |  |
| D3 |  |  |  |  |  |  |  |  |  |  |  |
| E3 |  |  |  |  |  |  |  |  |  |  |  |
| F3 |  |  |  |  |  |  |  |  |  |  |  |
| G3 |  |  |  |  |  |  |  |  |  |  |  |
| H3 |  |  |  |  |  |  |  |  |  |  |  |
| I3 |  |  |  |  |  |  |  |  |  |  |  |
| J3 |  |  |  |  |  |  |  |  |  |  |  |

| Emoji sequences | Levels of pain | | | | | | Please rate each emoji sequence on a Likert scale, from 1 (“unsuitable”) to 2 (“slightly suitable”) to 3 (“moderate suitable”) to 4 (“suitable”) to 5 (“very suitable”) based on your feelings about to what extent the emoji sequence can represent the pain levels from “no hurt” to “hurts most”. | | | | |
| --- | --- | --- | --- | --- | --- | --- | --- | --- | --- | --- | --- |
|  | 0 | 2 | 4 | 6 | 8 | 10 |  |  |  |  |  |
|  | No hurt | Hurts Little Bit | Hurts Little More | Hurts Even More | Hurts Whole Lot | Hurts Worst | unsuitable | slightly suitable | moderate suitable | suitable | very suitable |
| A4 |  |  |  |  |  |  |  |  |  |  |  |
| B4 |  |  |  |  |  |  |  |  |  |  |  |
| C4 |  |  |  |  |  |  |  |  |  |  |  |
| D4 |  |  |  |  |  |  |  |  |  |  |  |
| E4 |  |  |  |  |  |  |  |  |  |  |  |
| F4 |  |  |  |  |  |  |  |  |  |  |  |
| G4 |  |  |  |  |  |  |  |  |  |  |  |
| H4 |  |  |  |  |  |  |  |  |  |  |  |
| I4 |  |  |  |  |  |  |  |  |  |  |  |
| J4 |  |  |  |  |  |  |  |  |  |  |  |

Appendix: Emoji Faces (emoji 13.1 from Apple iOS 14.5)

|  |  |  |  |  |  |  |
| --- | --- | --- | --- | --- | --- | --- |
| A1 | A2 | A3 | A4 | A5 | A6 | A7 |
|  |  |  |  |  |  |  |
| B1 | B2 | B3 | B4 | B5 | B6 | B7 |
|  |  |  |  |  |  |  |
| C1 | C2 | C3 | C4 | C5 | C6 | C7 |
|  |  |  |  |  |  |  |
| D1 | D2 | D3 | D4 | D5 | D6 | D7 |
|  |  |  |  |  |  |  |
| E1 | E2 | E3 | E4 | E5 | E6 | E7 |
|  |  |  |  |  |  |  |
| F1 | F2 | F3 | F4 | F5 | F6 | F7 |
|  |  |  |  |  |  |  |
| G1 | G2 | G3 | G4 | G5 | G6 | G7 |
|  |  |  |  |  |  |  |
| H1 | H2 | H3 | H4 | H5 | H6 | H7 |
|  |  |  |  |  |  |  |
| I1 | I2 | I3 | I4 | I5 | I6 | I7 |
|  |  |  |  |  |  |  |
| J1 | J2 | J3 | J4 | J5 | J6 | J7 |
|  |  |  |  |  |  |  |
| K1 | K2 | K3 | K4 | K5 | K6 | K7 |
|  |  |  |  |  |  |  |
| L1 | L2 | L3 | L4 | L5 | L6 | L7 |
|  |  |  |  |  |  |  |
| M1 | M2 | M3 | M4 | M5 | M6 | M7 |
|  |  |  |  |  |  |  |
| N1 |  |  |  |  |  |  |

Emoji Faces (Google Android 11.0 December 2020 Feature Drop)

|  |  |  |  |  |  |  |
| --- | --- | --- | --- | --- | --- | --- |
| A1 | A2 | A3 | A4 | A5 | A6 | A7 |
|  |  |  |  |  |  |  |
| B1 | B2 | B3 | B4 | B5 | B6 | B7 |
|  |  |  |  |  |  |  |
| C1 | C2 | C3 | C4 | C5 | C6 | C7 |
|  |  |  |  |  |  |  |
| D1 | D2 | D3 | D4 | D5 | D6 | D7 |
|  |  |  |  |  |  |  |
| E1 | E2 | E3 | E4 | E5 | E6 | E7 |
|  |  |  |  |  |  |  |
| F1 | F2 | F3 | F4 | F5 | F6 | F7 |
|  |  |  |  |  |  |  |
| G1 | G2 | G3 | G4 | G5 | G6 | G7 |
|  |  |  |  |  |  |  |
| H1 | H2 | H3 | H4 | H5 | H6 | H7 |
|  |  |  |  |  |  |  |
| I1 | I2 | I3 | I4 | I5 | I6 | I7 |
|  |  |  |  |  |  |  |
| J1 | J2 | J3 | J4 | J5 | J6 | J7 |
|  |  |  |  |  |  |  |
| K1 | K2 | K3 | K4 | K5 | K6 | K7 |
|  |  |  |  |  |  |  |
| L1 | L2 | L3 | L4 | L5 | L6 | L7 |
|  |  |  |  |  |  |  |
| M1 | M2 | M3 | M4 | M5 | M6 | M7 |
|  |  |  |  |  |  |  |
| N1 |  |  |  |  |  |  |

Emoji Faces (Microsoft Windows 10 May 2019 Update)

|  |  |  |  |  |  |  |
| --- | --- | --- | --- | --- | --- | --- |
| A1 | A2 | A3 | A4 | A5 | A6 | A7 |
|  |  |  |  |  |  |  |
| B1 | B2 | B3 | B4 | B5 | B6 | B7 |
|  |  |  |  |  |  |  |
| C1 | C2 | C3 | C4 | C5 | C6 | C7 |
| NA |  |  |  |  |  |  |
| D1 | D2 | D3 | D4 | D5 | D6 | D7 |
|  |  |  |  |  |  |  |
| E1 | E2 | E3 | E4 | E5 | E6 | E7 |
|  |  |  |  |  |  |  |
| F1 | F2 | F3 | F4 | F5 | F6 | F7 |
|  |  |  |  |  |  |  |
| G1 | G2 | G3 | G4 | G5 | G6 | G7 |
|  |  |  |  |  |  |  |
| H1 | H2 | H3 | H4 | H5 | H6 | H7 |
|  |  |  |  | **NA** |  |  |
| I1 | I2 | I3 | I4 | I5 | I6 | I7 |
|  |  |  |  |  |  |  |
| J1 | J2 | J3 | J4 | J5 | J6 | J7 |
|  |  |  |  |  |  |  |
| K1 | K2 | K3 | K4 | K5 | K6 | K7 |
|  |  |  |  |  |  |  |
| L1 | L2 | L3 | L4 | L5 | L6 | L7 |
|  |  |  |  |  |  |  |
| M1 | M2 | M3 | M4 | M5 | M6 | M7 |
|  |  |  |  |  |  |  |
| N1 |  |  |  |  |  |  |

Emoji Faces (OpenMoji 13.0)

|  |  |  |  |  |  |  |
| --- | --- | --- | --- | --- | --- | --- |
| A1 | A2 | A3 | A4 | A5 | A6 | A7 |
|  |  |  |  |  |  |  |
| B1 | B2 | B3 | B4 | B5 | B6 | B7 |
|  |  |  |  |  |  |  |
| C1 | C2 | C3 | C4 | C5 | C6 | C7 |
|  |  |  |  |  |  |  |
| D1 | D2 | D3 | D4 | D5 | D6 | D7 |
|  |  |  |  |  |  |  |
| E1 | E2 | E3 | E4 | E5 | E6 | E7 |
|  |  |  |  |  |  |  |
| F1 | F2 | F3 | F4 | F5 | F6 | F7 |
|  |  |  |  |  |  |  |
| G1 | G2 | G3 | G4 | G5 | G6 | G7 |
|  |  |  |  |  |  |  |
| H1 | H2 | H3 | H4 | H5 | H6 | H7 |
|  |  |  |  |  |  |  |
| I1 | I2 | I3 | I4 | I5 | I6 | I7 |
|  |  |  |  |  |  |  |
| J1 | J2 | J3 | J4 | J5 | J6 | J7 |
|  |  |  |  |  |  |  |
| K1 | K2 | K3 | K4 | K5 | K6 | K7 |
|  |  |  |  |  |  |  |
| L1 | L2 | L3 | L4 | L5 | L6 | L7 |
|  |  |  |  |  |  |  |
| M1 | M2 | M3 | M4 | M5 | M6 | M7 |
|  |  |  |  |  |  |  |
| N1 |  |  |  |  |  |  |

Round 2 Survey Questionnaire (demographics forms omitted).

| Emoji sequences | Levels of pain | | | | | | Please rate each emoji sequence on a Likert scale, from 1 (“unsuitable”) to 2 (“slightly suitable”) to 3 (“moderate suitable”) to 4 (“suitable”) to 5 (“very suitable”) based on your feelings about to what extent the emoji sequence can represent the pain levels from “no hurt” to “hurts most”. | | | | |
| --- | --- | --- | --- | --- | --- | --- | --- | --- | --- | --- | --- |
|  | 0 | 2 | 4 | 6 | 8 | 10 |  |  |  |  |  |
|  | No hurt | Hurts Little Bit | Hurts Little More | Hurts Even More | Hurts Whole Lot | Hurts Worst | unsuitable | slightly suitable | moderate suitable | suitable | very suitable |
| A1 |  |  |  |  |  |  |  |  |  |  |  |
| B1 |  |  |  |  |  |  |  |  |  |  |  |
| C1 |  |  |  |  |  |  |  |  |  |  |  |
| D1 |  |  |  |  |  |  |  |  |  |  |  |

| Emoji sequences | Levels of pain | | | | | | Please rate each emoji sequence on a Likert scale, from 1 (“unsuitable”) to 2 (“slightly suitable”) to 3 (“moderate suitable”) to 4 (“suitable”) to 5 (“very suitable”) based on your feelings about to what extent the emoji sequence can represent the pain levels from “no hurt” to “hurts most”. | | | | |
| --- | --- | --- | --- | --- | --- | --- | --- | --- | --- | --- | --- |
|  | 0 | 2 | 4 | 6 | 8 | 10 |  |  |  |  |  |
|  | No hurt | Hurts Little Bit | Hurts Little More | Hurts Even More | Hurts Whole Lot | Hurts Worst | unsuitable | slightly suitable | moderate suitable | suitable | very suitable |
| A2 |  |  |  |  |  |  |  |  |  |  |  |
| B2 |  |  |  |  |  |  |  |  |  |  |  |
| C2 |  |  |  |  |  |  |  |  |  |  |  |
| D2 |  |  |  |  |  |  |  |  |  |  |  |

| Emoji sequences | Levels of pain | | | | | | Please rate each emoji sequence on a Likert scale, from 1 (“unsuitable”) to 2 (“slightly suitable”) to 3 (“moderate suitable”) to 4 (“suitable”) to 5 (“very suitable”) based on your feelings about to what extent the emoji sequence can represent the pain levels from “no hurt” to “hurts most”. | | | | |
| --- | --- | --- | --- | --- | --- | --- | --- | --- | --- | --- | --- |
|  | 0 | 2 | 4 | 6 | 8 | 10 |  |  |  |  |  |
|  | No hurt | Hurts Little Bit | Hurts Little More | Hurts Even More | Hurts Whole Lot | Hurts Worst | unsuitable | slightly suitable | moderate suitable | suitable | very suitable |
| A3 |  |  |  |  |  |  |  |  |  |  |  |
| B3 |  |  |  |  |  |  |  |  |  |  |  |
| C3 |  |  |  |  |  |  |  |  |  |  |  |
| D3 |  |  |  |  |  |  |  |  |  |  |  |

| Emoji sequences | Levels of pain | | | | | | Please rate each emoji sequence on a Likert scale, from 1 (“unsuitable”) to 2 (“slightly suitable”) to 3 (“moderate suitable”) to 4 (“suitable”) to 5 (“very suitable”) based on your feelings about to what extent the emoji sequence can represent the pain levels from “no hurt” to “hurts most”. | | | | |
| --- | --- | --- | --- | --- | --- | --- | --- | --- | --- | --- | --- |
|  | 0 | 2 | 4 | 6 | 8 | 10 |  |  |  |  |  |
|  | No hurt | Hurts Little Bit | Hurts Little More | Hurts Even More | Hurts Whole Lot | Hurts Worst | unsuitable | slightly suitable | moderate suitable | suitable | very suitable |
| A4 |  |  |  |  |  |  |  |  |  |  |  |
| B4 |  |  |  |  |  |  |  |  |  |  |  |
| C4 |  |  |  |  |  |  |  |  |  |  |  |
| D4 |  |  |  |  |  |  |  |  |  |  |  |
