# Supplementary material for: Development of the Emoji Faces Pain Scale and Its Validation on Mobile Devices in Adult Surgery Patients: Longitudinal Observational Study
Source: J Med Internet Res. 2023 Apr 17;25:e41189. doi: 10.2196/41189 (PMC10152337; doi:10.2196/41189)
Supplement: Multimedia Appendix 2 [file jmir_v25i1e41189_app2.docx]

# Multimedia Appendix 2. Supplemental methods and result.

## Development of Emoji-FPS

### Description of Delphi panelists’ characteristics

**Table S1. Characteristics of Delphi panelists**

| Characteristics | Results |
| --- | --- |
| Gender, n (%) |  |
| Male | 15 (37.5) |
| Age, mean (range), years | 37.4 (23-52) |
| Profession, n (%) |  |
| Anesthetist | 33 (82.5) |
| Nurse anesthetist | 6 (15.0) |
| Scientific research staff | 1 (2.5) |
| With intermediate qualification or above  for practicing pain medicine, n (%) | 10 (25.0) |
| Education level, n (%) |  |
| Below bachelor's degree | 1 (2.5) |
| Bachelor's degree | 13 (32.5) |
| Master's degree | 23 (57.5) |
| Doctor's degree | 3 (7.5) |
| Professional title, n (%) |  |
| Primary | 10 (25.0) |
| Intermediate | 23 (57.5) |
| Senior | 7 (17.5) |
| Working experience, mean (range), years | 13.7 (2-29) |
| Hospital location, n (%) |  |
| Shanghai, China | 36 (90.0) |
| Jiangsu Province, China | 3 (7.5) |
| Sichuan Province, China | 1 (2.5) |
| Degree of awareness of emoji, n (%) |  |
| Extremely aware | 3 (7.5) |
| Very aware | 13 (32.5) |
| Moderately aware | 17 (42.5) |
| Slightly aware | 3 (7.5) |
| Not all aware | 4 (10) |

### Results of Delphi Round 1 (n=40)

**Table S2. Results of Delphi survey Round 1**

| Type | Emoji-FPS  sequence | Score,  mean (SD) | Rank | Type | Emoji-FPS  sequence | Score,  mean (SD) | Rank |
| --- | --- | --- | --- | --- | --- | --- | --- |
| iOS | A | 2.45 (1.34) | 10 | Microsoft | A | 2.70 (1.16) | 10 |
|  | B | 3.10 (1.28) | 7 |  | B | 3.00 (1.13) | 9 |
|  | C | 3.33 (1.19) | 4 |  | C | 3.43 (1.08) | 4 |
|  | D | 4.08 (0.94) | 2 |  | D | 3.98 (0.83) | 3 |
|  | E | 4.15 (0.83) | 1 |  | E | 4.08 (0.80) | 1 |
|  | F | 3.33 (0.94) | 5 |  | F | 3.40 (0.84) | 6 |
|  | G | 4.05 (0.96) | 3 |  | G | 4.05 (0.90) | 2 |
|  | H | 3.23 (0.97) | 6 |  | H | 3.43 (0.98) | 5 |
|  | I | 3.03 (0.95) | 9 |  | I | 3.30 (0.91) | 7 |
|  | J | 3.03 (0.92) | 8 |  | J | 3.30 (0.88) | 8 |
| Android | A | 2.60 (1.19) | 10 | OpenMoji | A | 2.53 (1.20) | 10 |
|  | B | 3.03 (1.12) | 9 |  | B | 3.00 (1.13) | 9 |
|  | C | 3.25 (1.21) | 7 |  | C | 3.38 (1.19) | 6 |
|  | D | 4.00 (0.82) | 2 |  | D | 3.88 (0.88) | 1 |
|  | E | 4.05 (0.88) | 1 |  | E | 3.83 (0.84) | 3 |
|  | F | 3.48 (0.88) | 4 |  | F | 3.25 (1.01) | 7 |
|  | G | 3.98 (0.83) | 3 |  | G | 3.83 (1.03) | 2 |
|  | H | 3.38 (0.90) | 5 |  | H | 3.43 (0.90) | 4 |
|  | I | 3.15 (1.03) | 8 |  | I | 3.13 (1.04) | 8 |
|  | J | 3.28 (0.93) | 6 |  | J | 3.40 (0.93) | 5 |

### Results of Delphi Round 2 (n=36)

**Table S3. Results of Delphi survey Round 2**

| Type | Emoji-FPS  sequence | Score,  mean (SD) | Rank | Type | Emoji-FPS  sequence | Score,  mean (SD) | Rank |
| --- | --- | --- | --- | --- | --- | --- | --- |
| iOS | A | 3.94 (0.75) | 2 | Microsoft | A | 3.89 (0.85) | 2 |
|  | B | 4.08 (0.84) | 1 |  | B | 4.06 (0.75) | 1 |
|  | C | 3.92 (0.81) | 3 |  | C | 3.78 (0.96) | 3 |
|  | D | 3.78 (0.83) | 4 |  | D | 3.75 (0.91) | 4 |
| Android | A | 3.92 (0.65) | 2 | OpenMoji | A | 3.78 (0.83) | 3 |
|  | B | 4.03 (0.74) | 1 |  | B | 3.92 (0.84) | 1 |
|  | C | 3.92 (1.00) | 2 |  | C | 3.81 (0.95) | 2 |
|  | D | 3.89 (0.89) | 3 |  | D | 3.67 (0.89) | 4 |

## Validation of Emoji-FPS

### Description of design of validation study with Latin square table

**Supplemental methods.**

In the validation study, patients were asked to complete a questionnaire comprised of five scales each time, and they need to complete the questionnaire for five times. The occurrence order of each scale at each time point might have influence on measured results. Besides, we would like to randomly allocate all participants to five groups (A, B, C, D, and E) to ensure that patients would receive different types of questionnaires at each time point.

To achieve this purpose, we firstly designed five types of questionnaires, and the occurrence order of each scale was different between groups (See Table S4.1).

**Table S4.1. Questionnaire types.**

|  | Type of sequence | | | | |
| --- | --- | --- | --- | --- | --- |
| Sequence | S1 | S2 | S3 | S4 | S5 |
| 1 | WB FACES | VAS | FPS-R | NRS | Emoji-FPS |
| 2 | FPS-R | Emoji-FPS | NRS | VAS | WB FACES |
| 3 | Emoji-FPS | NRS | WB FACES | FPS-R | VAS |
| 4 | NRS | WB FACES | VAS | Emoji-FPS | FPS-R |
| 5 | VAS | FPS-R | Emoji-FPS | WB FACES | NRS |

Abbreviations: NRS, numerical rating scale; VAS, visual analogue scale; WB FACES, Wong-Baker FACES®; FPS-R, Faces Pain Scale-Revised; Emoji-FPS, Emoji faces pain scale.

The next step was to distribute the five types of questionnaires to a 5*5 table (5 time points * 5 groups). Here Latin square design was used to ensure that each type of questionnaire will occur only once in each column and row (See Table S4.2).

**Table S4.2. Questionnaire sequence by group ^a^.**

|  | Group | | | | |
| --- | --- | --- | --- | --- | --- |
| Time points | A | B | C | D | E |
| T1 | S1 | S2 | S4 | S5 | S3 |
| T2 | S2 | S3 | S1 | S4 | S5 |
| T3 | S3 | S5 | S2 | S1 | S4 |
| T4 | S4 | S1 | S5 | S3 | S2 |
| T5 | S5 | S4 | S3 | S2 | S1 |

^a^ 5*5 Latin square table was applied to the table.

Here another problem arose. We designed to compare agreement between four types of Emoji-FPS (iOS, Android, and Microsoft platforms and Openmoji). However, appearance of those our types of Emoji-FPS are very close, so we would like each of those Emoji-FPS types appearing only once at each timepoint. In order to test concurrent validity between types of Emoji-FPS, we only allow them to occur simultaneously at T4. For other time points (T1, T2, T3, and T5), only one type of Emoji-FPS would occur. Participants of group E would receive only iOS type of Emoji-FPS and was excluded from the Latin square table. Eventually, a 4*4 Latin square table was used to distribute four types of Emoji-FPS (See Table S4.3).

**Table S4.3. Occurrence order of Emoji-FPS by group ^a, b^.**

|  | Group | | | | |
| --- | --- | --- | --- | --- | --- |
| Time points | A | B | C | D | E |
| T1 | OpenMoji | iOS | Microsoft | Android | iOS |
| T2 | Microsoft | Android | OpenMoji | iOS | iOS |
| T3 | iOS | OpenMoji | Android | Microsoft | iOS |
| T4 | All | All | All | All | All |
| T5 | Android | Microsoft | iOS | OpenMoji | iOS |

Abbreviation: Emoji-FPS, Emoji faces pain scale. ^a^ 4*4 Latin square table was applied to the shaded cells. ^b^ iOS, Android, and Microsoft and Openmoji present four types of Emoji-FPS.

Based on above, the final full design matrix was as follow (Table S4.4).

**Table S4.4. Final design matrix ^a^.**

|  | Group | | | | |
| --- | --- | --- | --- | --- | --- |
| Time points | A | B | C | D | E |
| T1 | WB FACES | VAS | NRS | Android | FPS-R |
|  | FPS-R | iOS | VAS | WB FACES | NRS |
|  | OpenMoji | NRS | FPS-R | VAS | WB FACES |
|  | NRS | WB FACES | Microsoft | FPS-R | VAS |
|  | VAS | FPS-R | WB FACES | NRS | iOS |
| T2 | VAS | FPS-R | WB FACES | NRS | iOS |
|  | Microsoft | NRS | FPS-R | VAS | WB FACES |
|  | NRS | WB FACES | OpenMoji | FPS-R | VAS |
|  | WB FACES | VAS | NRS | iOS | FPS-R |
|  | FPS-R | Android | VAS | WB FACES | NRS |
| T3 | FPS-R | OpenMoji | VAS | WB FACES | NRS |
|  | NRS | WB FACES | Android | FPS-R | VAS |
|  | WB FACES | VAS | NRS | Microsoft | FPS-R |
|  | VAS | FPS-R | WB FACES | NRS | iOS |
|  | iOS | NRS | FPS-R | VAS | WB FACES |
| T4 | NRS | WB FACES | All Emoji-FPS | FPS-R | VAS |
|  | VAS | FPS-R | WB FACES | NRS | All Emoji-FPS |
|  | FPS-R | All Emoji-FPS | VAS | WB FACES | NRS |
|  | All Emoji-FPS | NRS | FPS-R | VAS | WB FACES |
|  | WB FACES | VAS | NRS | All Emoji-FPS | FPS-R |
| T5 | Android | NRS | FPS-R | VAS | WB FACES |
|  | WB FACES | VAS | NRS | OpenMoji | FPS-R |
|  | VAS | FPS-R | WB FACES | NRS | iOS |
|  | FPS-R | Microsoft | VAS | WB FACES | NRS |
|  | NRS | WB FACES | iOS | FPS-R | VAS |

Abbreviations: NRS, numerical rating scale; VAS, visual analogue scale; WB FACES, Wong-Baker FACES®; FPS-R, Faces Pain Scale-Revised; Emoji-FPS, Emoji faces pain scale.

^a^ iOS, Android, and Microsoft and Openmoji present four types of Emoji-FPS.

### Appearance of scales on questionnaire.

**Table S5. Questions used on mobile devices.**

| Type of pain scale | Questions |
| --- | --- |
| VAS | 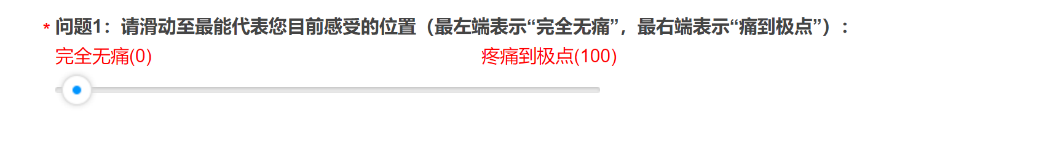 |
| NRS | 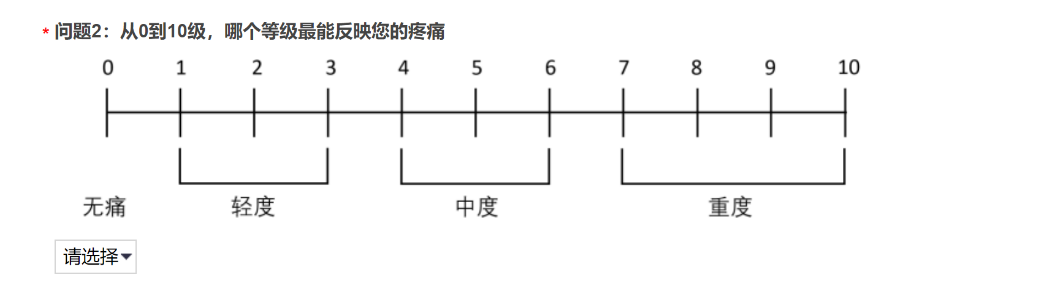 |
| FPS-R | 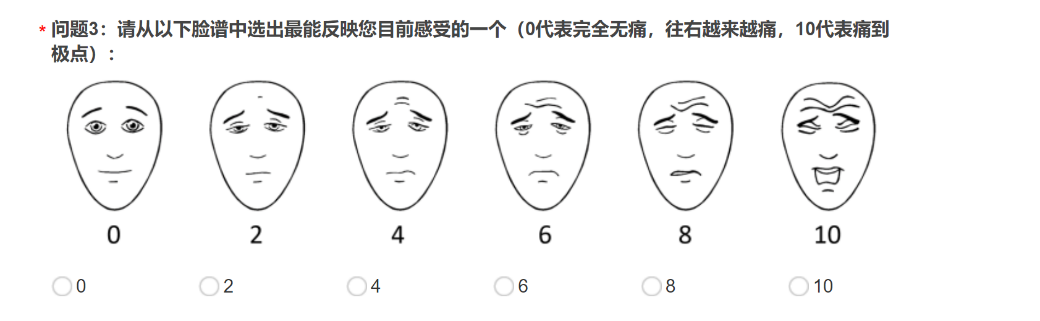 |
| WB FACES | 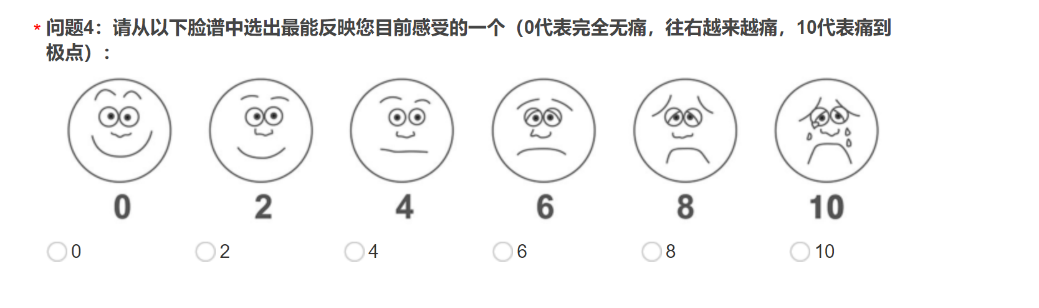 |
| Emoji-FPS (iOS) | 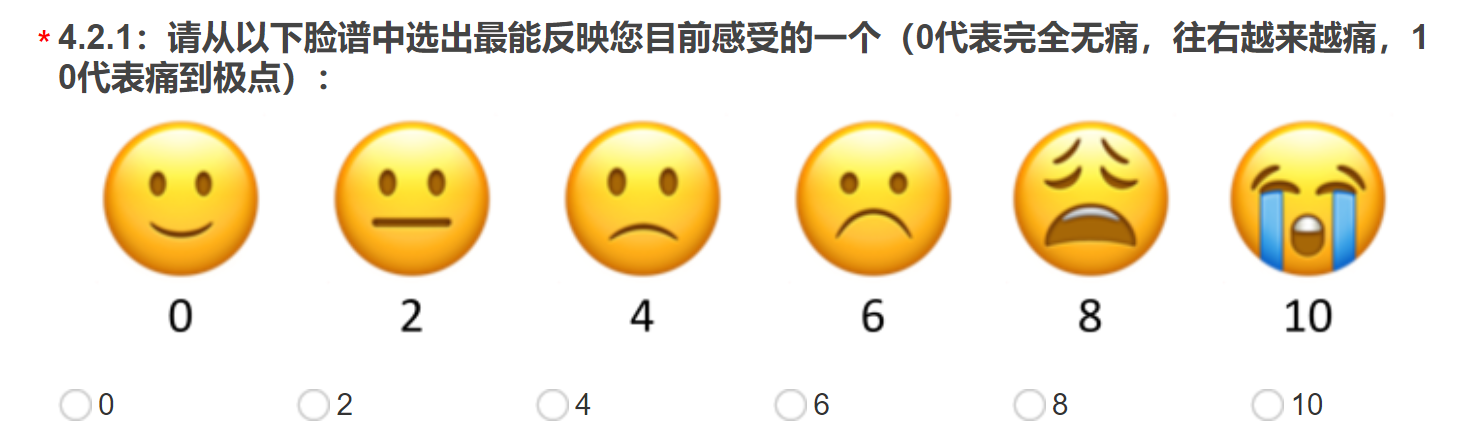 |
| Emoji-FPS (Android) | 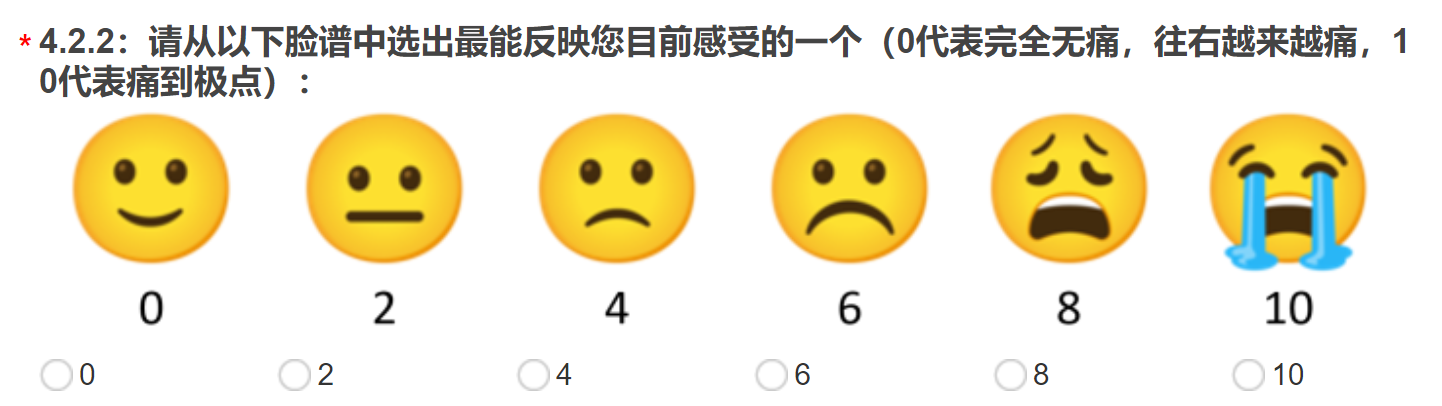 |
| Emoji-FPS (Microsoft) | 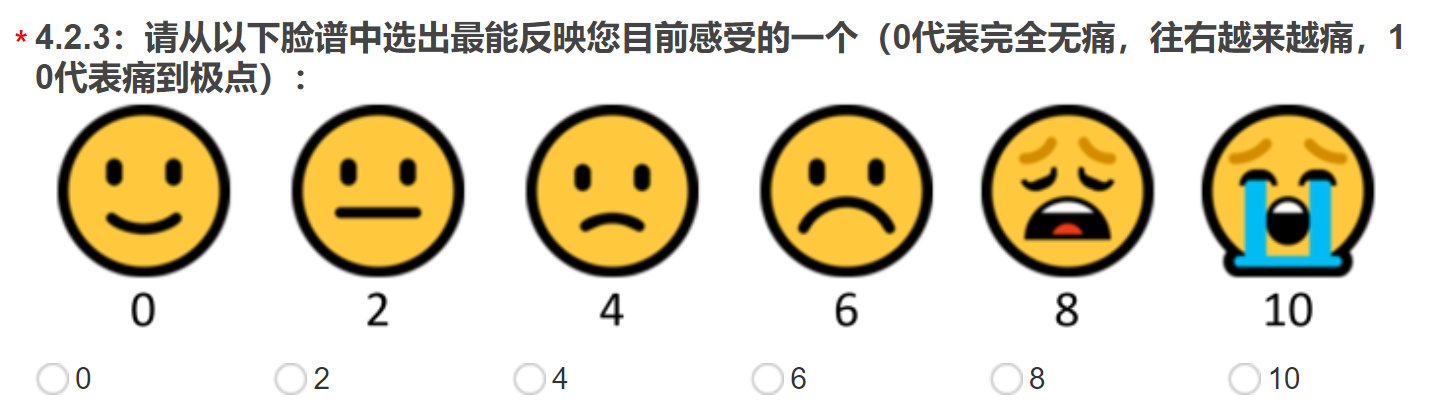 |
| Emoji-FPS (OpenMoji) | 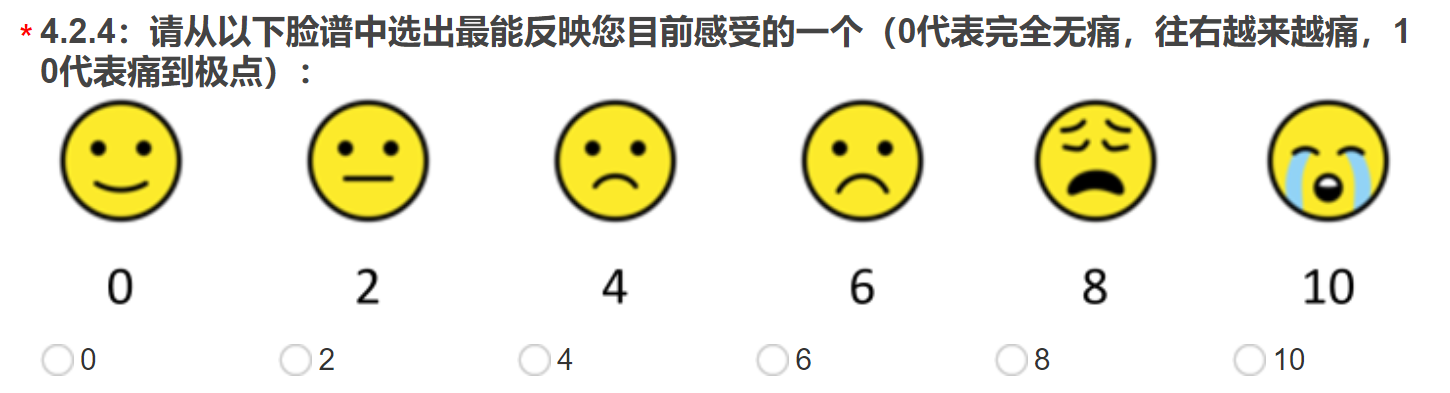 |

### Flow chart of the prospective observational validation study of Emoji Faces Pain Scale


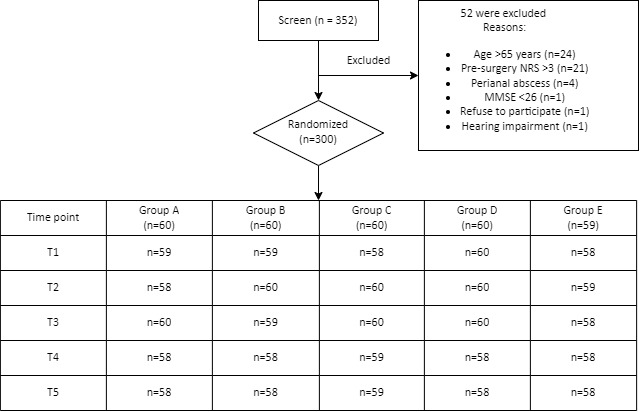


**Figure S1. Flow chart of the validation study.**

Abbreviations: NRS, numerical rating scale; MMSE, mini-mental state examination.

One patient was excluded from Group E due to pre-surgery NES >3.

### Description of study population of the validation study of Emoji Faces Pain Scale

**Table S6. Description of study population of the validation study.**

|  | Group | | | | |  |
| --- | --- | --- | --- | --- | --- | --- |
| Characteristics | A | B | C | D | E | Total |
| n | 60 | 60 | 60 | 60 | 59 | 299 |
| Sex, n(%) |  |  |  |  |  |  |
| Male | 37(61.7) | 39(65.0) | 38(63.3) | 39(65.0) | 40(67.8) | 193(64.6) |
| Female | 23(38.3) | 21(35.0) | 22(36.7) | 21(35.0) | 19(32.2) | 106(35.5) |
| Age, mean (SD), years | 42.2(12.3) | 35.7(9.7) | 38.0(9.8) | 40.0(12.2) | 36.7(6.7) | 38.5(10.5) |
| BMI, mean (SD), kg/m^2^ | 23.9(3.2) | 24.0(3.0) | 24.5(3.8) | 23.5(3.0) | 24.6(3.3) | 24.1(3.3) |
| Educational level, n(%) |  |  |  |  |  |  |
| High school or below | 24(40.0) | 14(23.3) | 19(31.7) | 23(38.3) | 14(23.7) | 94(31.4) |
| College or above | 36(60.0) | 46(76.7) | 41(68.3) | 37(61.7) | 45(76.3) | 205(68.6) |
| Surgery type, n(%) |  |  |  |  |  |  |
| Hemorrhoidectomy | 45(75.0) | 35(58.3) | 41(68.3) | 39(65.0) | 41(69.5) | 201(67.2) |
| Anal fistula resection | 15(25.0) | 25(41.7) | 19(31.7) | 21(35.0) | 18(30.5) | 98(32.8) |
| Surgery duration, mean (SD), minutes | 24.4(9.0) | 28.5(12.4) | 24.6(8.2) | 25.0(10.3) | 26.9(14.3) | 25.9(11.1) |

### Agreements between difference versions of Emoji-FPS

**Table S7. Concurrent validity between types of Emoji faces pain scale.**

|  | *Weighted Kappa* | | | |
| --- | --- | --- | --- | --- |
| Emoji-FPS type | iOS | Android | Microsoft | OpenMoji |
| iOS | 1 | NA | NA | NA |
| Android | 0.95 | 1 | NA | NA |
| Microsoft | 0.95 | 0.96 | 1 | NA |
| OpenMoji | 0.95 | 0.97 | 0.97 | 1 |

### Distribution of Emoji-FPS score over time.


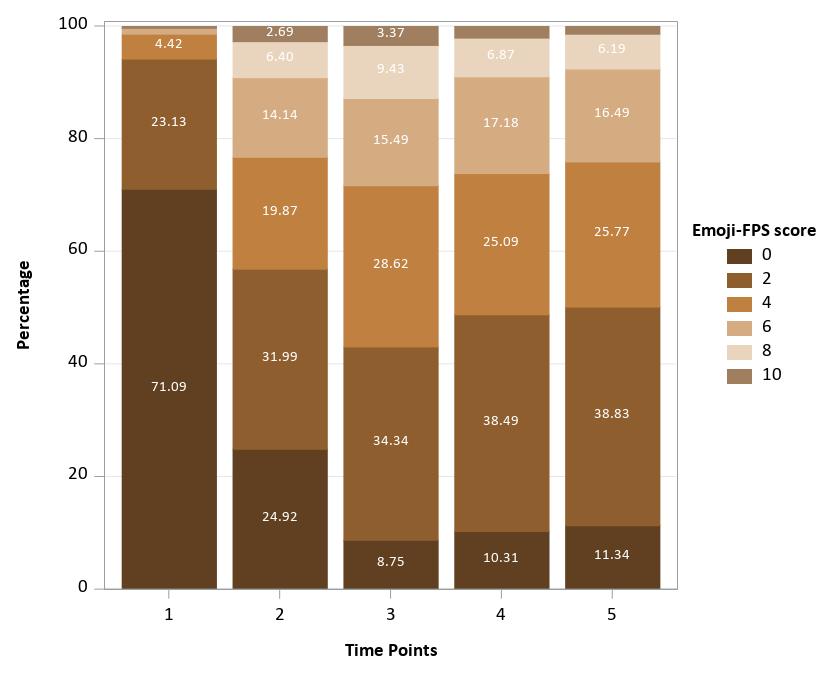


**Figure S2. Distribution of Emoji-FPS score over time.**
